# The antimicrobial systems of *Streptococcus suis* promote niche competition in pig tonsils

**DOI:** 10.1080/21505594.2022.2069390

**Published:** 2022-04-28

**Authors:** Zijing Liang, Huizhen Wu, Chen Bian, Hao Chen, Yanling Shen, Xueping Gao, Jiale Ma, Huochun Yao, Liping Wang, Zongfu Wu

**Affiliations:** a MOE Joint International Research Laboratory of Animal Health and Food Safety, College of Veterinary Medicine, Nanjing Agricultural University, Nanjing, China; b Key Lab of Animal Bacteriology, Ministry of Agriculture, Nanjing, China; c OIE Reference Lab for Swine Streptococcosis, Nanjing, China

**Keywords:** *Streptococcus suis*, lactococcin, lantibiotic bacteriocin, type VII secretion system, multidrug-resistant, bacterial competition

## Abstract

*Streptococcus suis* can cause severe infections in pigs and humans. The tonsils of pigs are major niches for *S. suis*, and different serotypes of *S. suis* can be found in the same tonsil. Pig tonsil colonization by *S. suis* is believed to be an important source of infection for humans and pigs. However, how *S. suis* competes for a stable tonsil niche is unknown. Here, we found that *S. suis* strain WUSS351, isolated from a healthy pig tonsil, is virulent and multidrug-resistant. The ABC transporter system SstFEG, conferring resistance to bacitracin, was reported to confer a competitive survival advantage *in vivo*. In addition, strain WUSS351 has several antimicrobial systems, including a novel type VII secretion system (T7SS), lantibiotic bacteriocin, and lactococcin972-like bacteriocin Lcn351. Bacterial competition experiments demonstrated T7SS-mediated cell contact-dependent antagonism of *S. suis*. Antibacterial activity analysis and 16S rRNA gene sequencing of the culture-independent and culture-dependent pig tonsillar microbiome revealed that Lcn351 mainly targets *S. suis*, one of the core microbiomes in pig tonsils. Taken together, our results revealed the mechanism of the stable persistence of *S. suis* in the tonsil niche, which might have important implications for *S. suis* epidemiology, potentially influencing strain prevalence and disease progression.

## Introduction

*Streptococcus suis* is able to cause meningitis, septicemia, arthritis, and sudden death in pigs [1]. *S. suis* is also pathogenic toward humans, acting as a zoonotic agent and infecting via contact with contaminated by-products or infected pigs [2,3]. In addition, *S. suis* promotes antimicrobial resistance by spreading resistance genes to other streptococci [4]. *S. suis* is a heterogeneous bacterial species. To date, 29 serotypes have been identified according to the antigenicity of their capsular polysaccharides (CPS). In addition, 28 novel *cps* loci (NCL) genotypes have been found recently [5–9]. Compared with the 29 serotypes, the information on NCL strains is very limited. The pig tonsil is colonized by many microbes, and it is a reservoir niche for some important bacterial pathogens, such as *S. suis* [10,11]. In fact, different serotypes of *S. suis* can be found from the same tonsil [12]. *S. suis* strains isolated from healthy pig tonsils can be pathogenic [3]. Liang *et al*. reported that four representative strains of *S. suis* serotype 7 isolated from healthy pig tonsils were identified as virulent strains; two of them belonged to ST373, the same sequence type with serotype 7 human isolate GX69, suggesting we should be aware of the public health threat posed by *S. suis* serotype 7 ST373 strains [13]. Wang *et al*. reported the pathogenicity of three representative strains of *S. suis* serotype 31 isolated from healthy pig tonsils [14]. Thus, infections in susceptible pigs [15] and humans [16,17] might derive from *S. suis* in the tonsils of healthy pigs.

In Gram-positive bacteria, the type VII secretion system (T7SS) and bacteriocins are important antimicrobial systems that mediate bacterial competition and infection. T7SS is specific secretion machinery for achieving contact-dependent toxins transport to target prey bacteria [18] and plays an essential role in virulence [19,20] and colonization [21,22] of pathogenic bacteria. Bacteriocins are antimicrobial effectors which play a critical role in bacterial competition to ﬁt its environmental niche [23,24]. However, we do not know how *S. suis* competes for a stable tonsil niche. The present study reveals the complete genome sequence of *S. suis* NCL4 strain WUSS351, a virulent and multidrug-resistant strain isolated from a healthy pig tonsil. In addition, this strain has several antimicrobial systems, including T7SS, lantibiotic bacteriocin, and lactococcin972-like bacteriocin Lcn351, which together promote its niche competition in pig tonsils. These findings contribute to understanding how *S. suis* stably persists in the tonsil niche and help to develop novel ways to control the disease caused by this bacterium.

## Materials and methods

### Bacterial strains and culture conditions

S. *suis* strain WUSS351 was isolated from a healthy pig tonsil in Jiangsu province, China. Table S1 provides detailed information on the strains and plasmids. *S. suis* strains were cultured in Todd-Hewitt broth (THB, Becton Dickinson, Franklin Lakes, NJ, USA) or on 6% (vol/vol) sheep blood-containing agar medium, at 37°C with 5% CO_2_. For *S. suis* mutant selection, the medium contained 100 μg/mL spectinomycin (Spc, Sigma-Aldrich, St. Louis, MO, USA), or 5 μg/mL chloramphenicol (Chl, Sigma-Aldrich, St. Louis, MO, USA). Luria-Bertani (LB, Becton Dickinson) medium was used to culture *Escherichia coli* strains at 37°C. To select bacteria harboring recombinant plasmids, 100 μg/mL ampicillin (Amp, Sigma-Aldrich) or 50 μg/mL Spc were added when necessary.

### Genome sequencing and bioinformatic analysis

A Bacterial DNA Kit (OMEGA Biotech, Norcross, GA, USA) was used to extract the genomic DNA from *S. suis* strain WUSS351, following the manufacturer’s guidelines. Novogene Bioinformatics Technology Co., Ltd (Beijing, China) used the Illumina NovaSeq PE150 system (Illumina, San Diego, CA, USA) to sequence the genomic DNA. SOAP *denovo*, version 2.04 [25] was used to carry out the *de novo* genome assembly. We deposited the genome sequence and annotation for strain WUSS351 at NCBI (Accession No. NZ_CP039462.1). Possible genomic islands (GIs) in the genome were predicted using Island Viewer 4 [26]. BLAST algorithms at the NCBI were used to perform homology analysis. The genetic distance among *S. suis* strains was calculated using the OrthoANIu algorithm [27]. Based on the distance data, a phylogenetic tree by the Neighbor-Joining method was generated with MEGA X software [28]. Multiple alignments of genomes were carried out using the Mauve software [29], and the Circos software was used to display the comparison results [30].

### Transmission electron microscopy (TEM)

The capsule of *S. suis* was observed using TEM using a previously published method [6]. A JEM-1010 TEM (JEOL, Ltd., Tokyo, Japan) was used to examine the capsule of *S. sui*s following the supplier’s protocol.

### Models of animal infection

Experimental infection of animals was performed in the Laboratory Animal Center of Nanjing Agricultural University with the approval of the institution’s ethics committee (Permit number SYXK (Su) 2017–0007). The zebraﬁsh infection model detailed in previous studies was used [14,17,31]. Exponentially grown *S. suis* strains were rinsed three times using phosphate-buffered saline (PBS) and adjusted to the indicated doses. Zebraﬁsh (n = 15 per group) were received an intraperitoneal injection of 10-fold serially diluted suspensions containing 10^5^–10^8^ colony forming units (CFU) of bacteria. For 7 days post-infection, the mortality of the fish was recorded. The Reed and Muench method [32] was used to calculate the lethal dose (LD)_50_. A highly virulent *S. suis* strain, serotype 2 meningitis isolate SC070731, was used as a positive control [14,33]. A BALB/c mouse infection model was also used to further evaluate the virulence of strain WUSS351. Ten mice per group were injected intraperitoneally with 3 × 10^8^ CFU/mouse for each strain, and mouse mortality was monitored for 7 days.

### Construction of the gene deletion mutants

Deletion mutant strains Δ*T7SS* and Δ*lcn351* were constructed via natural transformation using our previously published protocol [34]. The upstream and downstream of deletion region fragments were obtained by PCR assay from WUSS351 genomic DNA employing primers Δ*T7SS-*A/B and Δ*T7SS-*C/D, or Δ*lcn351-*A/B and Δ*lcn351-*C/D. Primers *spc*-F/R or *chl*-F/R were used to amplify the *spc* or *chl* gene. Fusion PCR with primers Δ*T7SS-*A/D or Δ*lcn351-*A/D was used to ligate the three amplification products. One microgram of the fusion fragment and the ComS13–21 peptide (DFSTSWWNF, 250 μM) (ChinaPeptides Co., Ltd., Shanghai, China) were mixed with 100 μL of strain WUSS351 (OD_600_ = 0.04–0.06) and incubated statically at 37°C for 2 h. After that, the mixture was spread on THB Spc or THB Chl agar and incubated at 37°C. Single colonies were selected and subjected to PCR to detect the deletion mutant using primers Δ*T7SS-*E/F or Δ*lcn351-*E/F. Sequencing then confirmed the deletion mutant. Table S2 lists the PCR primers used.

### Testing antimicrobial susceptibility

Minimum inhibitory concentrations (MICs) of the following antimicrobials toward strain WUSS351 were determined by the broth microdilution method: rifampin, β-lactam (cefotaxime, amoxicillin, and penicillin), oxazolidinone (linezolid), glycopeptide (vancomycin), fluoroquinolones (enrofloxacin), amphenicols (florfenicol and chloramphenicol), lincosamides (lincomycin and clindamycin), aminoglycosides (gentamycin and spectinomycin), macrolides (tilmicosin, tulathromycin, erythromycin, and azithromycin), tetracyclines (doxycycline), and polypeptide (bacitracin) as described in our previous study [14]. The breakpoints with resistant values were determined based on our previous study [14].

### RNA extraction and quantitative real-time reverse transcription PCR (RT-qPCR)

The bacterial RNA was extracted using a FastRNA Pro Blue Kit (MP biomedicals, Santa Anna, CA, USA). Our previous study [31] describes the detailed procedure for qRT-PCR analysis. Briefly, a reverse transcription kit (Vazyme, Nanjing, China) was used to produce cDNA from the RNA. The quantitative real-time PCR (qPCR) step, using the cDNA as the template, was carried out on a QuantStudio 6 Flex apparatus (Thermo Fisher Scientific, Shanghai, China) using a SYBR Premix Ex Taq^TM^ (Takara) kit following the supplier’s protocols. Table S2 lists the specific primers used for RT-qPCR. The transcript level of the *rpoB* gene was constant under all conditions; therefore, its transcription level was used to normalize that of the target genes. The 2^−ΔΔCT^ method was used to calculate the relative fold change. Results are shown as the mean ± the standard error of the mean (SEM) of three independent experiments.

### Western blotting analysis

The strain WUSS351 in the mid-log phase was subjected to centrifugation at 8,000 × *g* at 4°C for 15 min. For the whole-cell proteins, PBS was used to wash the bacterial pellet three times, which was then suspended in 1× SDS-PAGE sample loading buﬀer and boiled for 5 min. Secreted proteins were extracted according to our previously published protocol [35]. SDS-PAGE was used to separate the proteins in the samples, followed by transfer via semi-dry blotting (GE Healthcare, Chicago, IL, USA) onto PVDF membranes using a TE77 instrument (GE Healthcare). 5% skimmed milk in PBS containing 0.05% Tween-20 (PBST) was then used to block the membranes for 2 h at 37°C. Anti-FLAG antibodies recognizing Sequential Peptide Affinity (SPA)-tagged proteins [36] (Sigma) or anti-GroEL antibodies [35] were incubated with the membranes, followed by three washes with PBST buﬀer, and then incubated with horseradish peroxidase (HRP)-conjugated anti-rabbit secondary antibody (Boster, Wuhan, China). Finally, the immunoreactive proteins on the membranes were detected using an ECL Western blotting kit (Tanon, Shanghai, China) on a Tanon 5100 instrument.

### Tonsillar microbiota analysis by 16S rRNA gene sequencing

Four tonsils collected from four healthy pigs were minced, weighed, and suspended at 1:9 (wt/vol) in sterile PBS. DNA was extracted directly from the tonsils for culture-independent analysis using the cetyltrimethylammonium bromide method [37] and sent to Novogene Bioinformatics Technology Co., Ltd. for 16S rRNA gene sequencing. For culture-dependent analysis, tonsil samples were then processed for 80 seconds at a setting of 6.0 in a Homogenizer (MP biomedicals) to release the bacteria from the tonsillar matrix. According to a previous study [10], six different media were used to cultivate a wide variety of bacteria under aerobic or anaerobic conditions. The tonsil homogenate of tonsils was added into the following six media at a ratio of 1:50 (vol/vol): Columbia broth (Hopebio, Qingdao, China), Tryptone soybean broth (TSB, Hopebio), MacConkey (Hopebio), THB, Gifu anaerobic medium (GAM, Hopebio), and Brain heart infusion (Hopebio) supplemented with 10 mg/mL each of hemin and NAD (abbreviated as BHIXV). The synthesized 73-aa Lcn351 peptide (20 μg/mL), corresponding to the putative mature form of Lcn351, (AVQYPDGGVWTYGASNGGAFSNYYHGKKEHSSTVVSRKDSRSAKGSAGPGQTSKAYIKTSFGEPAAFYYDFWQ) (ChinaPeptides Co., Ltd.) was added into the above media as the Lcn351 groups, and for the control group, an equal volume of H_2_O was added. The media with the tonsil homogenate, including Columbia broth, TSB, MacConkey, THB, and BHIXV, were incubated aerobically at 37°C for 8 h with 5% CO_2_, and the GAM medium with homogenate was incubated anaerobically for 8 h at 37°C. Bacteria were collected by centrifugation at 8,000 × *g* for 10 min, washed two times with PBS, resuspended, and mixed together from the six media for each tonsil. The DNA extracted from mixed bacteria was sent to Novogene Bioinformatics Technology Co., Ltd. for 16S rRNA gene sequencing. Sequences reads were grouped into operational taxonomic units (OTUs) at a sequence similarity level of ≥97% for a taxonomic assignment using Uparse software (http://drive5.com/uparse/) [38,39], and species annotation was conducted through the SSUrRNA Database at the Silva Database (https://www.arb-silva.de/). Subsequent alpha and beta diversity analyses were performed based on the normalized data. The relative abundance of OTUs was represented as the mean ± SEM.

### Culture with supernatant or the Lcn351 peptide

To culture different bacterial targets in the presence of strain WUSS351 or Δ*lcn351* supernatants, the supernatants of overnight (10 h) culture (strains WUSS351 or Δ*lcn351*, 25 mL) were centrifuged at 13,000 × *g*. The supernatants were filtered through a 0.22 mm pore size filter (BIOFIL, Indore, India). Bacterial targets (10^7^ cells) were inoculated at 37°C into 2.5 mL of strain WUSS351 or Δ*lcn351* supernatants and 2.5 mL of fresh medium: nutrient broth (Hopebio) for *Bacillus subtilis*; MRS broth (Hopebio) for *Lactobacillus lactis* and *Lactobacillus rhamnosus*; LB for *E. coli*; THB for *S. suis*, *S. dysgalactiae*, *Streptococcus equi* subsp. *zooepidemicus*, *Streptococcus agalactiae*, *Aeromonas hydrophila*, and *Klebsiella pneumoniae*. At 6 h after inoculation, the cultures were serially diluted and plated on the appropriate agar plates. For *in vitro* assays, 10^7^ bacterial cells were inoculated into different concentrations of the synthesized Lcn351 peptide (10 or 20 μg/mL) into 5 mL of the medium at 37°C as test groups. For the control groups, an equal volume of H_2_O was added. At 6 h after inoculation, the cultures were serially diluted and plated. The results were calculated as the relative growth rate (RGR) = AGR _Lcn351 or H2O_ /AGR _Lcn351_, where AGR represents the absolute growth rate = CFU _final_ /CFU _initial_. Data are expressed as the mean ± SEM.

### Bacterial contact-dependent competition experiments

Similar to the rifampin (Rif)-resistant induced strain *S. suis* P1/7-Rif [40], *L. lactis* strain WUQT017, and *S. dysgalactiae* strain WUQT020 were selected using increasing amounts of antibiotics to produce a Rif-resistant phenotype. The stepwise induction method was used to produce nalidixic acid (Nal)-resistant phenotypes of *A. hydrophila* strain WUQT018, *K. pneumoniae* strain WUQT019, and *E. coli* strain WUQT022. For the competition assays, the attacker and prey strains were diluted to an initial OD_600_ of 0.6 using THB. The attacker and prey cells were mixed at a ratio of 10:1 (CFU _attacker_ : CFU _prey_), centrifuged, and resuspended in 50 μL of PBS. Two microliters of the mixture were spotted on LB agar medium and grown for different times at 37°C with 5% CO_2_ for Gram-positive bacteria. The mixture was spotted onto a nitrocellulose membrane on LB agar medium for the competition experiments for Gram-negative bacteria. The CFU of the prey and attacker strains was calculated to determine the starting ratio. The competition was determined by excising the agar with cell growth and resuspending the cells in 1 mL PBS. The final count of the prey was calculated by plating serial dilutions onto appropriate agar plates with selective antibiotics. The information related to the antibiotics and agar plates for the prey was as follows: *S. suis* strain P1/7-Rif and *S. dysgalactiae* strain WUQT020-Rif, 8 μg/mL Rif and THB agar plates; *S. suis* strain Δ*T7SS*, 100 μg/mL Spc and THB agar plates; *L. lactis* strain WUQT017-Rif, 8 μg/mL Rif and MRS agar plates; *A. hydrophila* strain WUQT018-Nal, 32 μg/mL Nal and TSB agar plates; *K. pneumoniae* strain WUQT019-Nal, 32 μg/mL Nal and MacConkey agar plates; *E. coli* strain WUQT022-Nal, 32 μg/mL Nal and LB agar plates.

### Statistical analysis

The Log-rank (Mantel-Cox) test was used to analyze the survival curve in the animal experiments. A two-tailed paired *t* test was used to compare the Lcn351 and control groups. A two-tailed unpaired *t* test was used for the rest of the experiments. GraphPad Prism software was used to perform all the statistical analyses.

## Results

### WUSS351 belongs to the NCL4 type and is an encapsulated and virulent strain

**Figure 1.**
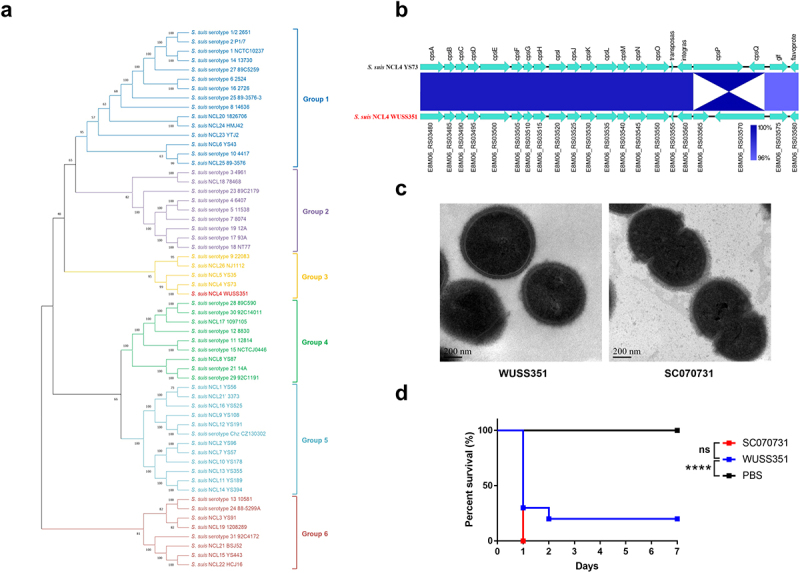
**The capsule and pathogenicity of strain WUSS351**. (a) The phylogenetic tree of *S. suis cps* clusters from different serotypes or genotypes, created using the Neighbor-Joining method. (b) The comparative analysis of *cps* gene clusters between *S. suis* NCL4 strains WUSS351 and YS73. (c) The capsule of *S. suis* strains WUSS351 and SC070731 (an encapsulated strain control), as observed using TEM. (d) The survival curve of mice injected with strains WUSS351 and SC070731 (a virulent strain control) at the dose of 3 × 10^8^ CFU/mouse or PBS (a negative control). Asterisks indicate pairs of significantly different values (n = 10, the Log-rank (Mantel-Cox) test, **** indicates p < 0.0001, ns indicates not significant).

Strain WUSS351 has a genome comprising a single circular chromosome of 2,283,286 bp, but no plasmids. Table S3 summarizes the general genomic features. Table S4 lists the 39 predicted genomic islands (GIs). To determine the *cps* genotype of strain WUSS351, phylogenetic tree analysis based on the sequence of the *cps* cluster in all *S. suis* strains (29 serotypes and 28 NCL types) was performed, which showed that strain WUSS351 is located at Group 3, together with NCL4 strain YS73, NCL5 strain YS35, serotype 9 strain 22083, and NCL26 strain NJ1112 (Figure 1a). The sequence of *cps* cluster of strain WUSS351 shared 98.86% nucleotide identity with that of NCL4 strain YS73 (GenBank: KM972278.1) (Figure 1b), confirming that strain WUSS351 belongs to the NCL4 type. Transmission electron microscopy (TEM) analysis showed that strains WUSS351 and SC070731 (as a positive control) [6] were encapsulated (Figure 1c).

The zebrafish infection model was first used to evaluate the virulence of strain WUSS351. The LD_50_ value of strain WUSS351 was 1.1 × 10^6^ CFU/fish, similar to that of the virulent control strain SC070731 [14,33] (8.6 × 10^5^ CFU/fish). Then, their virulence was further determined in a mouse infection model. As shown in Figure 1d, strains WUSS351 and SC070731 induced 80% and 100% mortality, respectively, and a Log-rank (Mantel-Cox) test showed that their virulence was not significantly different.

### Strain WUSS351 is multidrug-resistant

To determine which antimicrobials strain WUSS351 is susceptible to, a MIC assay was performed for 19 antimicrobials comprising 11 classes. WUSS351 showed resistance to nine antimicrobials of five classes (Table 1). A strain that is resistant to ≥ 3 classes of antimicrobials is considered multidrug-resistant [41]. Thus, strain WUSS351 is multidrug-resistant. A BLAST search for acquired antimicrobial resistance genes in strain WUSS351 was performed to clarify the antibiotic resistance mechanisms. In addition to the macrolide-lincosamide-streptogramin B resistance gene *erm*(B) and the tetracycline resistance gene *tet*(O), other antimicrobial resistance genes were found, including an ABC transporter system gene *sstFEG* and Bce system genes *bceBA* and *bceSR*, which confer bacitracin resistance in *S. suis* [42]. The genes *erm*(B) and *tet*(O) were in predicted GIs 19 and 20, respectively (Table 1), while the bacitracin resistance genes *sstFEG*, *bceBA*, and *bceSR* were in an integrative and conjugative element (ICE) (ICE_WUSS351_) (Figure S1). We observed a chromosomal mutation of *pbp2x*, which is involved in resistance to penicillin [43]. Thus, the resistance phenotype is in accordance with the genotype of antimicrobial resistance.

**Table 1.** MICs of strain WUSS351

Classes	Antibiotics	Breakpoints for resistant (mg/L)	MICs (mg/L)	Phenotype	Resistance mechanisms	Inside of MGEs
β-lactam	Penicillin	≥1	2	R	87.33% identity of PBP2X to P1/7	
	Amoxicillin	>2	≤0.0625			
	Cefotaxime	≥8	0.5			
Rifamycins	Rifampin	>0.5	≤0.0625			
Glycopeptide	Vancomycin	>1	1			
Oxazolidinone	Linezolid	>2	2			
Fluoroquinolones	Enrofloxacin	≥2	1			
Amphenicols	Chloramphenicol	≥16	4			
	Florfenicol	≥8	4			
Lincosamides	Lincomycin	≥1	>256	R	*erm*(B)	No.19 GI
	Clindamycin	≥1	>256	R	*erm*(B)	No.19 GI
Aminoglycosides	Gentamycin	≥16	4			
	Spectinomycin	≥128	32			
Macrolides	Tilmicosin	≥32	>256	R	*erm*(B)	No.19 GI
	Tulathromycin	≥64	>256	R	*erm*(B)	No.19 GI
	Erythromycin	≥1	>256	R	*erm*(B)	No.19 GI
	Azithromycin	≥2	>256	R	*erm*(B)	No.19 GI
Tetracyclines	Doxycycline	≥2	32	R	*tet*(O)	No.20 GI
Polypeptide	Bacitracin	-	>256	R	*BceB/A*, *BceS/R*, *sstFEG*	ICE_WUSS351_

MGEs, Mobile genetic elements; GI, genomic island; R, resistance.

### Strain WUSS351 harbors a novel T7SS that mediates contact-dependent antagonism of S. suis

**Figure 2.**
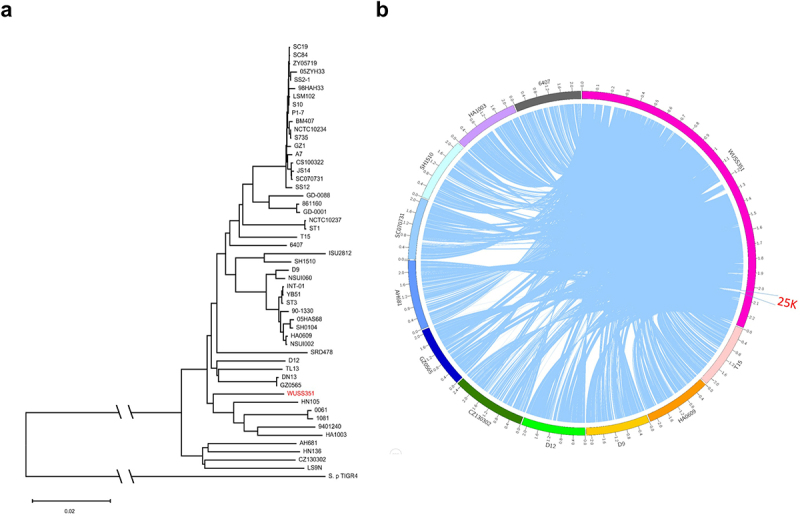
**Comparative genome analysis**. (a) The genetic distance among *S. suis* strains was calculated using the OrthoANIu algorithm. Based on the distance data, Neighbor-Joining method was used to construct the phylogenetic tree. *S. pneumoniae* strain TIGR4 was used as an out-group. (b) The Mauve software was used to align 52 *S. suis* genomes, and the Circos software was used to display comparison results. To better present the comparative genome analysis results, strain WUSS351 and representative strains from different branches of the phylogenetic tree shown in Figure 2a were selected. A 25 kb genomic island was predicted in the genome of strain WUSS351 but not in the other 51 *S. suis* strains.

To investigate the phylogenetic relationship between strain WUSS351 and 51 strains whose complete genomic sequences are available from NCBI, phylogenetic tree analysis based on whole genomic sequences was performed. As shown in Figure 2a, strain WUSS351 was closely related to *S. suis* serotype 4 strain HA1003, serotype Chz strain 0061, serotype 31 strain 1081, serotype 9 strain 9401240, and serotype 5 strain HN105, and distantly related to serotype 2 strains SC19, SC84, ZY05719, 05HAH33, SS2–1, 98HAH33, S10, P1/7, BM407, S735, GZ1, and SC070731.

**Figure 3.**
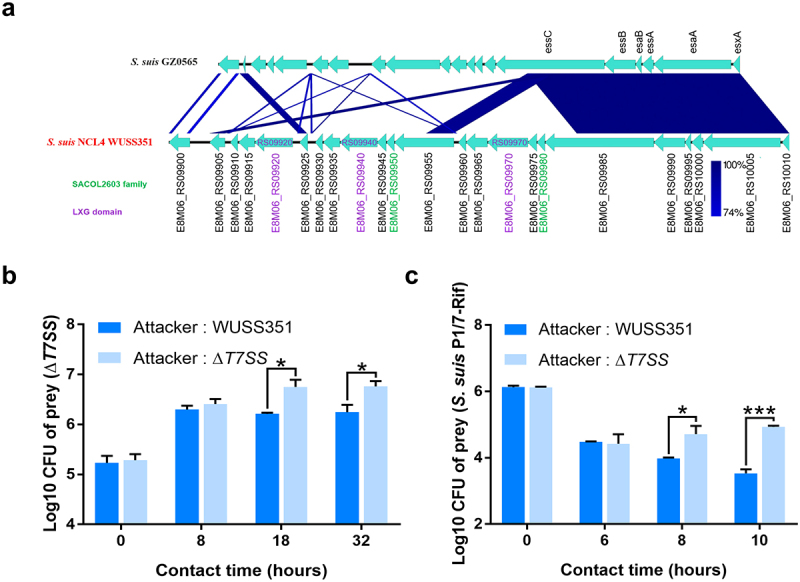
**The T7SS of strain WUSS351**. (a) Comparison of the sequence of the T7SS clusters between strains WUSS351 and GZ0565. (b) Results of competition experiments performed with WUSS351 or Δ*T7SS* as attacker strains and Δ*T7SS* as the prey strain. The number of the final prey cells was determined at 8, 18, and 32 h by serially diluting cultures onto LB+Spc agar plates. (c) Results of competition experiments performed with WUSS351 or Δ*T7SS* as attacker strains and *S. suis* strain P1/7-Rif as the prey strain. The number of the final prey cells was determined at 6, 8, and 10 h by serially diluting cultures onto LB+Rif agar plates. The data in the graphs represent the means ± SEM, and asterisks indicate pairs of significantly different values (n = 3, *** indicates *p* <0.001, * indicates *p* <0.05, two-tailed unpaired *t* test).

Comparative genomic analysis showed that strain WUSS351 contains a 25-kb GI that was not detected in the other 51 *S. suis* strains (Figure 2b). The 25-kb GI contains a novel T7SS cluster (Figure 3a), and several *S. suis* strains harbor a T7SS with 40–45% sequence similarity to this novel T7SS (Table S5). In Gram-positive bacteria, the T7SS mediates contact-dependent antagonism during bacterial competition [44], which motivated us to investigate the capacity of this novel T7SS to mediate inter-bacterial antagonism. As shown in Figure 3b, significant contact-dependent antagonism of Δ*T7SS* was observed in co-cultured with wild-type strain WUSS351 at 18 h and 32 h, compared with co-cultured with the strain lacking T7SS. A similar contact-dependent antagonism of *S. suis* virulent reference strain P1/7-Rif was observed when co-cultured with wild-type strain WUSS351 at 8 h and 10 h, compared with co-culture with the strain lacking the T7SS (Figure 3c). The wild-type strain and Δ*T7SS* showed no significant changes in growth in THB medium (Figure S2). However, the T7SS of WUSS351 did not exhibit its capacity of inter-bacterial antagonism when interacting with other five strains isolated from pig tonsils using six different media described in tonsillar microbiota analysis, consisting of *Lactococcus lactis* strain WUQT017-Rif, *Aeromonas hydrophila* strain WUQT018-Nal, *Klebsiella pneumoniae* strain WUQT019-Nal, *Streptococcus dysgalactiae* strain WUQT020-Rif, and *Escherichia coli* strain WUQT022-Nal (data not shown).

### Lactococcin Lcn351 inhibits the growth of Bacillus subtilis and S. suis

Further genomic analysis found that strain WUSS351 contains two different bacteriocins. One is a lantibiotic bacteriocin (encoded by *E8M06_RS05390*). The nucleotide sequence of this bacteriocin locus in strain WUSS351 shares 100% identity with that of *suicin3908* locus in *S. suis* 3908 [45] (Figure S3a). Western blotting analysis showed that strain WUSS351 could express this bacteriocin as a SPA-tagged fusion protein (Figure S3b). Vaillancourt *et al*. reported that Suicin3908 was resistant to high temperature, pH, and proteases digestion and was active against *S. suis* and *Staphylococcus hyicus* [45].

**Figure 4.**
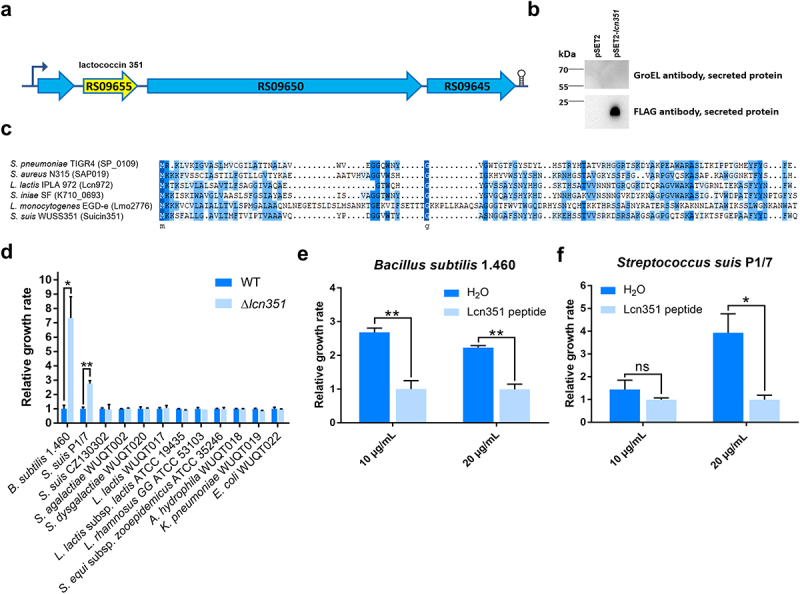
**Lcn351 targets *B. subtilis* and *S. suis***. (a) The genetic organization of the lactococcin cluster (blue) and Lcn351 (yellow) in strain WUSS351. (b) The secreted proteins extracted from strain WUSS351 expressing Lcn351 fused with a SPA tag from its own promoter of the cluster (pSET2-*lcn351*) or control plasmid pSET2 were probed with FLAG or GroEL antibodies. (c) Alignment of the amino acid sequences of Lcn972 family bacteriocins: SP_0109 of *S. pneumoniae* strain TIGR4, SAP019 of *S. aureus* strain N315, Lcn972 of *L. lactis* strain IPLA 972, K710_0693 of *S. iniae* strain SF, and Lmo2776 of *L. monocytogenes* strain EGD-e. (d) The relative growth rate of different bacteria calculated after 6 h of incubation with supernatants of WT or Δ*lcn351*. The relative growth rate of *B. subtilis* 1.460 (e) and *S. suis* P1/7 (f) after 6 h of incubation in the presence of Lcn351 peptide (10 or 20 mg/mL) or H_2_O; the relative growth rate was compared with Lcn351 group. The graph shows the means ± SEM; asterisks indicate pairs of significantly different values (n = 3, ** indicates *p* <0.01, * indicates *p* <0.05, ns indicates not significant, two-tailed unpaired *t* test).

The characteristics of Suicin3908 have been studied; therefore, we focused on the second bacteriocin in strain WUSS351, a lactococcin972-like bacteriocin (named as Lcn351, encoded by *E8M06_RS09655*) (Figure 4a). Western blotting analysis confirmed that strain WUSS351 could secrete Lcn351 as a SPA-tagged fusion protein (Figure 4b). As a control, GroEL, a cytoplasmic protein in *S. suis* [35], was not detected among the secreted proteins, reflecting no impurities in the secreted proteins from autolytic products. As shown in Figure 4c, the amino acid sequence of Lcn351 shares 37.63% identity with Lcn972 of *L. lactis* strain IPLA 972, 29.85% identity with Lmo2776 of *Listeria monocytogenes* strain EGD-e, 28.12% identity with SP_0109 of *Streptococcus pneumoniae* strain TIGR4, 35.42% identity with SAP019 of *Staphylococcus aureus* strain N315, and 40.62% identity with K710_0693 of *Streptococcus iniae* strain SF. The mRNA expression level of genes in *lcn351* locus was significantly higher in the stationary phase than in the exponential phase in THB (Figure S4). To determine the inhibitory spectrum of Lcn351, 12 strains were selected, consisting of six strains isolated from pig tonsils, three strains isolated from diseased pigs, and three strains that could be targets of the lactococcin972-like bacteriocins according to previous reports [23,46]. After incubating with the supernatant of wild-type (WT) or Δ*lcn351* from the stationary phase for 6 h, the growth of *B. subtilis* strain 1.460 and *S. suis* strain P1/7 was dramatically inhibited in the WT supernatant group compared with that in the Δ*lcn351* supernatant group (Figure 4d). However, Lcn351 did not affect the growth of the other ten strains. To further confirm the function of Lcn351, the activity of its synthesized peptide was analyzed on *B. subtilis* strain 1.460 and *S. suis* strain P1/7. Synthesized Lcn351 was observed to inhibit the growth of these two strains (Figure 4e, f).

### Lactococcin Lcn351 mainly targets S. suis in pig tonsils

**Figure 5.**
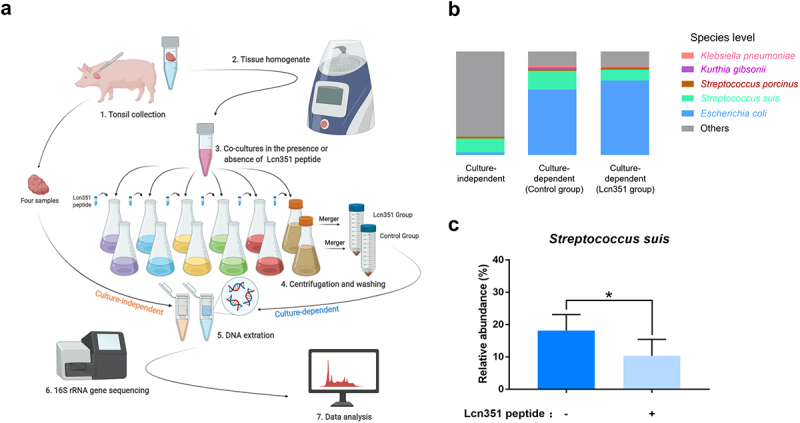
**Lcn351 targets *S. suis* in pig tonsils microbiota**. (a) A schematic diagram of the culture-independent and culture-dependent pig tonsillar microbiome analysis. Please refer to the Materials and methods section for the detailed protocols. (b) The relative abundance of species in the culture-independent and culture-dependent communities of pig tonsils. (c) The relative abundance of *S. suis* in the culture-dependent community of pig tonsils. The data in the graphs represent the means ± SEM, and asterisks indicate pairs of significantly different values (n = 4, * indicates p < 0.05, two-tailed unpaired *t* test).

To determine the target of Lcn351 in pig tonsils, the microbiota composition of pig tonsils was first verified by 16S rRNA gene sequencing. We found members of 15 phyla, 18 classes, 38 orders, 63 families, 122 genera, and 91 species of bacteria in one or more tonsils (Table S6) through sequencing the culture-independent tonsillar microbiome (Figure 5a). We found 12 of 15 phyla, 11 of 18 classes, 22 of 38 orders, 31 of 63 families, and 40 of 122 genera were also reported as pig tonsillar microbiota in a previous report [47]. The detailed list of pig tonsillar microbiota reported in both studies is summarized in Table S6. We defined the core microbiome of the pig tonsils as those presented in two or more samples and had a relative abundance exceeding 1% (Tables S6 and S7). Notably, the average relative abundance of *Streptococcus* genus was 14.13%, marking it as one member of the core microbiomes in pig tonsils in all tonsils (Table S6). Distribution at the species level followed well from the genus level data. *S. suis* was one of the dominant microbial communities in porcine tonsils, with an average relative abundance of 13.52% (Table S6), consistent with the previous study wherein *S. suis* was present in most pig tonsils [47].

Next, we compared the microbiota compositions cultured from pig tonsils with or without synthesized Lcn351 using 16S rRNA gene sequencing. According to a previous study [10], as shown in Figure 5a, we used six different media to cultivate various bacteria from pig tonsils under aerobic or anaerobic conditions. The cultured bacteria from each group were merged for 16S rRNA gene sequencing. As expected, the microbiota composition by culture-dependent analysis in the control group (without synthesized Lcn351) was significantly different from that obtained by culture-independent analysis (Figure S5a, b). The complete list of all taxons identified in the culture-dependent microbiome in the control group is summarized in Table S8. The most significant differences in microbiota composition included increased levels of *Enterobacteriaceae* (from 0.29% relative abundance by culture-independent analysis to 65.20% relative abundance by culture-dependent analysis) and *Staphylococcaceae* (from 0.42% relative abundance by culture-independent analysis to 9.13% relative abundance by culture-dependent analysis) (Figure S5a, b, and Tables S6 and S8). As shown in Figure 5b, compared with the culture-independent microbial community, *S. suis* was still one of the dominant bacteria in the culture-dependent microbial community, although *E. coli* became the most dominant bacteria. The culture-dependent microbiota composition of pig tonsils was dramatically altered after the addition of Lcn351. Species whose relative abundance exceeded 1% by culture-dependent analysis in the control group are presented in Figure 5b. The genera whose relative abundance exceeded 1% by culture-dependent analysis in the control group are presented in Figure S6a. The complete list of all taxons identified in the culture-dependent microbiome in the Lcn351 group is summarized in Table S9. Comparing the relative abundance at the genus level shown in Tables S8 and S9, the relative abundance of the *Streptococcus* genus was reduced from 23.08% to 13.15% after adding Lcn351, while that of the *Escherichia* genus increased from 63.22% to 72.13%. The reduced relative abundance of *Streptococcus* genus was mainly caused by the decreased relative abundance of *S*. *suis* (from 18.15% to 10.32%) (Figure 5c). In addition, the relative abundances of *S*. *porcinus*, *K. pneumoniae*, and *Kurthia gibsonii* also decreased in the group with Lcn351; however, their changes were not significant (Figure S6b-d). These findings indicated that *S. suis* is the primary target of Lcn351 in pig tonsils.

## Discussion

To date, the only available information for *S. suis* NCL4 was that it could be isolated from healthy pigs [7,48] and diseased pigs [8]. In this study, we found that *S. suis* NCL4 strain WUSS351 isolated from a healthy pig tonsil is virulent and has several systems that promote its niche competition in pig tonsils. Strain WUSS351 is multidrug-resistant, which might confer a competitive advantage to preferentially colonize the host over other microbiota constituents in the presence of antimicrobial selective pressures, leading to an increased prevalence. In addition, some bacteria from the host microflora can produce bacitracin [42]. Thus, strain WUSS351’s resistance to bacitracin might represent a competitive advantage in the host environment. Our previous study found that *sstFEG* is present mainly in *S. suis* virulent strains isolated from pigs or humans [42]. *sstFEG* not only confers resistance to bacitracin but also contributes to *S. suis* virulence via a competitive survival advantage *in vivo* [42].

T7SS is pivotal for pathogenic bacterial competitive capability and colonization. *S. aureus* utilizes its T7SS to secrete toxins that inhibit the growth of the rival bacteria, such as EsaD, causing DNA damage as a nuclease toxin [49], and TspA, targeting the periplasm of competitor bacteria as a membrane-depolarizing toxin [50]. The T7SS of *Streptococcus gallolyticus* subsp. *gallolyticus* contributes to its murine gut colonization by enhancing adherence capacity [21]. We first identified the T7SS in *S. suis* virulent strain GZ0565 and demonstrated that its substrate, EsxA, contributed to *S. suis* virulence [35]. However, the sequence coverage of this novel T7SS between strains WUSS351 and GZ0565 is only 42%. After comparing the T7SS cluster sequence with that of serotype 9 virulent strain GZ0565, strain WUSS351 was observed to harbor three LXG domain-containing proteins (with 34–58% identity, encoded by *E8M06_RS09920*, *E8M06_RS09940*, and *E8M06_RS09970*) in the T7SS (Figure 3a). The LXG domain is present in the *N*-terminus of a group of bacterial toxins predicted to be exported by the T7SS [51]. In addition to the *N*-terminal LXG domain, LXG family toxins usually contain a C-terminal variable toxin domain [51]. The three LXG-containing effectors of *Streptococcus intermedius* T7SS mediated inter-bacterial antagonism, and one of them degrades lipid II required for cell wall biosynthesis [52]. Additionally, the SACOL2603 family proteins are also considered as potential T7SS effectors and are similar in length and sequence to WXG family proteins [53]. Two SACOL2603 family proteins (encoded by *E8M06_RS09950* and *E8M06_RS09980*) were predicted (Figure 3a). Thus, it would be interesting to determine their functions in both virulence and bacterial competition in the future.

Many bacterial species produce small antimicrobial peptides termed bacteriocins, which are believed to function in inter-bacterial competition. The bacteriocin BlpC of *S. pneumoniae* contributes to competition and fitness by targeting neighboring bacteria for lysis [54]. The bacteriocin Nukacin IVK45 increases the competitive capability of *Staphylococcus epidermidis* in human nasal microbiotas [55]. *L. monocytogenes* bacteriocin LLS contributes to virulence and intestinal survival in a murine oral infection model, inhibits the growth of *L. monocytogenes, L. lactis*, and *S. aureus*, and promotes persistence in murine intestinal microbiota [56]. We found that strain WUSS351 possesses two bacteriocins. One is a lantibiotic bacteriocin whose nucleotide shares 100% identity with *suicin3908* of *S. suis* strain 3908. Suicin3908 has membrane permeabilization activity and shows antibacterial activity against ten pathogenic *S. suis* strains (members of the ST1, ST25, or ST28 groups) and *S. hyicus* [45], indicating that this bacteriocin might provide an inter-bacterial competitive advantage. The other bacteriocin is Lcn351, belonging to the lactococcin 972 family. Lactococcin Lcn972 of *L. lactis* subsp. *lactis* specifically kills other lactococci by inhibiting bacterial cell division via blocking the incorporation of cell wall precursors in the septum [46]. Lactococcin Lmo2776 of *L. monocytogenes* mainly targets the intestinal bacteria *Prevotella copri* to reduce its abundance and avoid the excessive inflammation caused by *P. copri*, which affects *L. monocytogenes* colonization in the host intestine [57]. Lactococcin Sil of *S. iniae* targets *B. subtilis*, inhibits host cell innate immune responses and increases bacterial infection by promoting dissemination and colonization [23]. Our study found lactococcin972-like bacteriocin Lcn351 mainly targeted *S. suis* based on the following reasons: 1) in antibacterial activity analysis, 12 strains mainly isolated from pig tonsils or diseased pigs were selected; in addition to *B. subtilis* strain 1.460 reported as a target of lactococcin972-like bacteriocins [23,57], Lcn351 only inhibited the growth of *S. suis* strain P1/7 (Figure 4d), not the other ten strains; 2) by 16S rRNA gene sequencing of the culture-dependent pig tonsillar microbiome, more than 22 species were identified; the significant change of the relative abundance after the addition of Lcn351 was only observed in *S. suis* (Tables S8 and S9), not the other species. We observed that Lcn351 inhibited the growth of *S. suis* in the tonsil. However, only two *S. suis* strains (serotype 2 strain P1/7 and serotype Chz strain CZ130302) were used in antibacterial activity analysis. Therefore, we did not know if this inhibition effect of Lcn351 was limited to a specific type or serotype of *S. suis*.

Inter-bacterial competition in the porcine tonsil during co-colonization might have important implications for the epidemiology of *S. suis*, potentially impacting serotype distributions, strain prevalence, and disease progression. Efficient removal of competitors could allow *S. suis* strain WUSS351 to colonize the tonsils more efficiently and for longer periods, which would increase its transmission potential. Currently, we are studying the correlation between the antimicrobial systems and *S. suis’* ability to colonize and induce disease in a larger panel of clinical isolates.

## Supplementary Material

Supplemental Material

## Data Availability

The complete genome sequence of strain WUSS351 was deposited in GenBank and received the accession number NZ_CP039462.1. The data supporting this study’s findings are available from the corresponding author upon reasonable request.
