# Supplementary material for: The antimicrobial systems of Streptococcus suis promote niche competition in pig tonsils
Source: Virulence. 2022 Apr 28;13(1):781–93. doi: 10.1080/21505594.2022.2069390 (PMC9067509; doi:10.1080/21505594.2022.2069390)
Supplement: Supplemental Material [file KVIR_A_2069390_SM0560.zip › supplementary/suppl.pdf]

Table S1. Summary of bacterial strains and plasmids

Strain or Plasmid	Description	Source or reference
**Bacterial strains**		
WUSS351	*S. suis* NCL4 genotype strain WUSS351 was isolated from the tonsil of a healthy pig in Jiangsu, China	This study
SC070731	*S. suis* setotype 2 virulent strain SC070731was isolated from a diseased pig in China	1
P1/7	Virulent SS2 of *S.suis* was isolated from a diseased pig in UK	2
P1/7-Rif	Strain P1/7 was induced to be rifampin resistant, Rif^r	3
Δ*T7SS* mutant	Deletion mutant of RS09900-RS010010 fragment with WUSS351 background, Spc^r	This study
Δ*lcn351* mutant	Deletion mutant of 1,972,041-1,975,272 fragment with WUSS351 background, Chl^r	This study
WUQT017	*L. lactis* strain WUQT017 was isolated from the tonsil of a healthy pig in China	This study
WUQT017-Rif	Strain WUQT017 was induced to be rifampin resistant, Rif^r	This study
WUQT018	*A. hydrophila* strain WUQT018 was isolated from the tonsil of a healthy pig in China	This study
WUQT018-Nal	Strain WUQT018 was induced to be nalidixic acid resistant, Nal^r	This study
WUQT019	*K. pneumoniae* strain WUQT019 was isolated from the tonsil of a healthy pig in China	This study
WUQT019-Nal	Strain WUQT019 was induced to be nalidixic acid resistant, Nal^r	This study
WUQT020	*S. dysgalactiae* strain WUQT020 was isolated from the tonsil of a healthy pig in China	This study
WUQT020-Rif	Strain WUQT020 was induced to be rifampin resistant, Rif^r	This study
WUQT022	*E. coli* strain WUQT022 was isolated from the tonsil of a healthy pig in China	This study
WUQT022-Nal	Strain WUQT022 was induced to be nalidixic acid resistant, Nal^r	This study
B. subtilis 1.460	*B. subtilis* 1.460 was purchased from China General Microbiological Culture Collection Center (CGMCC)	CGMCC
CZ130302	The serotype Chz strain *S. suis* CZ130302 was isolated from an acute piglet meningitis in eastern China	4
ATCC 35246	*S. equi* subsp. zooepidemicus ATCC35246 was isolated from a dead pig in China	5
WUQT002	*S. agalactiae* strain WUQT002 was isolated from the tonsil of a healthy pig in China	This study
ATCC 19435	*L. lactis* subsp. *lactis* ATCC 19435 was purchased from China General Microbiological Culture Collection Center (CGMCC)	CGMCC
ATCC 53103	*L. rhamnosus GG* ATCC 53103 was isolated from fecal samples of a healthy human	6
DH5α	Cloning host for maintaining the recombinant plasmids	Vazyme
Top10	Cloning host for maintaining the recombinant plasmids	Vazyme
**Plasmids**		

pSET-2	*S. suis* /*E. coli* shuttle vector, Spc^r	Daisuke Takamatsu
pSET-2-*suicin3908*	pSET-2 carrying RS05390 coding region (1-165aa) with SPA tag fusion with its original promoter, Spc^r	This study
pSET-2-*lcn351*	pSET-2 carrying 1,975,272-1,974,757 fragment with SPA tag fusion with the original promoter of the first gene of Lcn351 cluster, Spc^r	This study

Rif^r, rifampin-resistant phenotype. Nal^r, nalidixic acid-resistant phenotype. Spc^r, spectinomycin resistance cassette. Chl^r, chloramphenicol resistance cassette.

References:

[1] Wu, Z. F. et al. Comparative genomic analysis shows that *Streptococcus suis* meningitis isolate SC070731 contains a unique 105 K genomic island. Gene 535, 156-164, doi:10.1016/j.gene.2013.11.044 (2014).

[2] Holden, M. T. G. *et al.* Rapid Evolution of Virulence and Drug Resistance in the Emerging Zoonotic Pathogen *Streptococcus suis* . *Plos One* **4**, doi:10.1371/journal.pone.0006072 (2009).

[3] Huang, J. *et al.* Evolution and Diversity of the Antimicrobial Resistance Associated Mobilome in *Streptococcus suis* : A Probable Mobile Genetic Elements Reservoir for Other Streptococci. *Front Cell Infect Microbiol* **6**, 118, doi:10.3389/fcimb.2016.00118 (2016).

[4] Pan, Z. *et al.* Novel variant serotype of *streptococcus suis* isolated from piglets with meningitis. *Appl Environ Microbiol* **81**, 976-985, doi:10.1128/AEM.02962-14 (2015).

[5] Ma, Z. *et al.* Complete genome sequence of *Streptococcus equi* subsp. *zooepidemicus* strain ATCC 35246. *J Bacteriol* **193**, 5583-5584, doi:10.1128/JB.05700-11 (2011).

[6] Saxelin, M., Pessi, T. & Salminen, S. Fecal recovery following oral administration of *Lactobacillus* strain GG (ATCC 53103) in gelatine capsules to healthy volunteers. *Int J Food Microbiol* **25**, 199-203, doi:10.1016/0168-1605(94)00091-j (1995).

Table S2. Primers used in this study

Primer	Sequence (5’-3’)	Comment
**Construction of deletion strains**		
Δ*lcn351* -A	AACTTGCTGGCCTTGAGAAAATAAAC	Upstream of fusion fragment for Δ *lcn351* mutant
Δ*lcn351* -B	CCAATTGTCTAAATCAATTTTATTAAAGTTCATATGGAATTTAGTATTAAGTAAGAATTGAAAATC	
chl -F	ATGAACTTTAATAAAATTGATTTAGACAATTGG	Chloramphenicol resistance gene
chl -R	TTATAAAAGCCAGTCATTAGGCCTAT	
Δ*lcn351* -C	ATAGGCCTAATGACTGGCTTTTATAAGAATATCGTTTCCGTTCTTCCTTTTCTCCT	Downstream of fusion fragment for Δ *lcn351* mutant
Δ*lcn351* -D	TCATCGTAGAGATATTTATTAAAAGCTGGT	
Δ*lcn351* -E	GTTTCATTAGGTTACTCCTTTTCAT	Detection of deletion of *lcn351* gene
Δ*lcn351* -F	CGATTGAGATAATCCCCACAA	
Δ*T7SS* -A	TCGCATTCCCCAAGCCTTTCC	Upstream of fusion fragment for Δ *T7SS* mutant
Δ*T7SS* -B	CAAATATATCCTCCTCACTATTTATTTTGTTTAGATCATATTAG	
spc -F	AAATAGTGAGGAGGATATATTTG	Spectinomycin resistance gene
spc -R	TTTTTTTATAATTTTTTTAATCTGTTATTT	
Δ*T7SS* -C	AAATAACAGATTAAAAAAATTATAAAAAAAATCGTTCTCCTTTTGTCTTATAA	Downstream of fusion fragment for Δ *T7SS* mutant
Δ*T7SS* -D	ATGTAAGCGATAAACAAAATACG	
Δ*T7SS* -E	AATAAAACACTCTTCCCTCCG	Detection of deletion of *T7SS* gene
Δ*T7SS* -F	TATCTTTTTAGCAGTGGTCGC	
**Construction of expression vectors**		
Promotor-T7SS-A	AAAACGACGGCCAGTgaattcTGCTCTGCGGTCAAAATAGTA	Promotor of VII secretion system
Promotor-T7SS-B	ATCGTTCTCCTTTTGTCTTAT	
Promotor-Suicin3908-A	AAAACGACGGCCAGTgaattcAAAATTTAATAAGAGGAATAAAGAGAACT	Promotor of Suicin39008
Promotor-Suicin3908-B	ATTCATTATAAATTCCTCCTTGAATAATTAAA	
2S-Suicin3908-C	TTTAATTATTCAAGGAGGAATTTATAATGAATATTAAACCAGAAATCTATGTTCAAA	Fusion fragment for construction of pSET-2-*suicin3908*
2S-Suicin3908-D	TTCTCTTTTCCATGGAggatccTTAACAATTTTTGCATCCAGA	
Promotor-Lcn351-A	AAAACGACGGCCAGTgaattcAATCTACTCCTTTCAAAAAACTG	Promotor of Lcn351
Promotor-Lcn351-B	GAATATCGTTTCCGTTCTTCCTT	
2S-Lcn351-C	AAGGAAGAACGGAAACGATATTCTTGGTAGGAATTGAGGCATTC	Fusion fragment for construction of pSET-2-

2S-Lcn351-D	TTCTCTTTTCCATGGAggatccTTATTGCCAGA AATCATAATAAAATGCAG	construction of pSET-2-*lcn351*
pSET-2-F	TAAGTTGGGTAACGCCAGGG	The fragments inserted into pSET-2 for verification
pSET-2-R	ACACAACATACGAGCCGGAA	
**RT-qPCR**		
qRT-RS09645-F	AGGATGGAGTGGTTGAATATGG	The transcriptional level of RS09645 mRNA
qRT-RS09645-R	CGGTTCGTCTGCCAAGATTA	
qRT-RS09650-F	GGTCGTGAGGGTCTTGATATTG	The transcriptional level of RS09650 mRNA
qRT-RS09650-R	ACCAGCTAGACGACGGATAA	
qRT-RS09655-F	TCGCTTTACTTGGTGCTGTT	The transcriptional level of RS09655 mRNA
qRT-RS09655-R	TCCAGCAGATCCCTTAGCA	
qRT-rpoB-F	TCTTGGAAGCTGGTACTGTATTG	The transcriptional level of RS00745 mRNA as reference gene
qRT-rpoB-F	CTCAGCCAAGTTGAGGAAGTAG	

[a] Underlined nucleotides denote reverse complement; lowercase nucleotides denote restriction enzyme sites.

Table S3. Basic characteristics of strain WUSS351 genome

Size (bp)	GC%	CDS	rRNA	tRNA	GIs
2,283,286	41.39	2,196	12	55	39

Table S4. The predicted gene islands of strain WUSS351

Number	Start	End	Width
No.1	16,506	24,814	8,308
No.2	72,345	128,500	56,155
No.3	92,353	98,087	5,734
No.4	135,511	166,347	30,836
No.5	318,861	331,186	12,325
No.6	347,055	352,079	5,024
No.7	363,680	367,883	4,203
No.8	383,292	388,242	4,950
No.9	480,320	485,258	4,938
No.10	488,003	496,822	8,819
No.11	488,954	498,601	9,647
No.12	512,671	520,680	8,009
No.13	699,863	707,388	7,525
No.14	708,893	714,559	5,666
No.15	797,348	801,348	4,000
No.16	819,465	825,354	5,889
No.17	822,430	826,811	4,381
No.18	952,362	956,518	4,156
No.19	1,051,563	1,061,834	10,271
No.20	1,068,029	1,083,401	15,372
No.21	1,177,484	1,230,368	52,884
No.22	1,203,079	1,209,420	6,341
No.23	1,272,024	1,276,403	4,379
No.24	1,272,295	1,288,004	15,709
No.25	1,272,812	1,303,107	30,295
No.26	1,286,842	1,292,559	5,717
No.27	1,293,685	1,299,692	6,007
No.28	1,339,999	1,347,552	7,553
No.29	1,448,590	1,452,726	4,136
No.30	1,543,673	1,567,701	24,028
No.31	1,583,717	1,587,837	4,120
No.32	1,804,969	1,810,183	5,214
No.33	1,833,610	1,838,161	4,551
No.34	1,906,688	1,912,719	6,031
No.35	2,031,892	2,057,116	25,224
No.36	2,071,769	2,076,283	4,514
No.37	2,183,150	2,189,159	6,009
No.38	2,247,975	2,259,520	11,545
No.39	2,248,521	2,252,905	4,384

Table S5. Sequence alignment of the novel T7SS between WUSS351 and other strains

Strain	Identity	Query cover
Streptococcus suis strain GZ0565	99%	42%
Streptococcus suis strain DN13	99%	42%
Streptococcus suis strain ST3	99%	45%
Streptococcus suis strain D12	98%	40%
Streptococcus suis strain 05HAS68	99%	45%
Streptococcus suis strain NSUI002	99%	45%
Streptococcus suis strain YB51	99%	45%
Streptococcus suis strain HA0609	99%	45%

Table S6. Complete list of all taxon identified in culture-independent microbiome

Complete list of all phyla identified

**Phylum**[a]	**Tonsil.1**	**Tonsil.2**	**Tonsil.3**	**Tonsil.4**	**% of total**
Firmicutes*	17.94%	89.72%	30.09%	32.13%	42.47%
Bacteroidetes*	43.22%	4.93%	45.82%	22.21%	29.05%
Fusobacteria*	11.67%	3.19%	9.00%	35.78%	14.91%
Proteobacteria*	24.39%	0.80%	11.91%	7.82%	11.23%
Actinobacteria*	0.98%	0.00%	0.18%	0.18%	0.34%
Spirochaetes*	0.00%	0.16%	0.70%	0.26%	0.28%
Tenericutes*	0.14%	0.30%	0.48%	0.14%	0.27%
Cyanobacteria*	0.66%	0.00%	0.04%	0.08%	0.19%
Acidobacteria*	0.16%	0.00%	0.06%	0.08%	0.08%
Synergistetes*	0.00%	0.18%	0.00%	0.06%	0.06%
Gracilibacteria	0.12%	0.00%	0.00%	0.00%	0.03%
Planctomycetes*	0.06%	0.00%	0.00%	0.00%	0.01%
Chloroflexi*	0.06%	0.00%	0.00%	0.00%	0.01%
Nitrospinae	0.02%	0.00%	0.00%	0.00%	0.00%
Deinococcus-Thermus	0.02%	0.00%	0.00%	0.00%	0.00%
Others	0.56%	0.72%	1.72%	1.26%	1.07%

[a] Phyla belonging to the core microbiome are highlighted.

* Phyla have also been reported as pig tonsillar microbiota in a previous study (Lowe et al., BMC microbiology. 2012).

Complete list of all class identified

**Class**[b]	**Tonsil.1**	**Tonsil.2**	**Tonsil.3**	**Tonsil.4**	**% of total**
Bacteroidia*	43.22%	4.93%	45.82%	22.21%	29.05%
Clostridia*	8.30%	34.05%	24.67%	23.35%	22.59%
Bacilli*	9.00%	51.05%	3.05%	8.16%	17.82%
Fusobacteriia*	11.67%	3.19%	9.00%	35.78%	14.91%
Gammaproteobacteria*	23.47%	0.72%	10.56%	6.96%	10.43%
Negativicutes	0.32%	4.49%	2.22%	0.28%	1.83%
Alphaproteobacteria*	0.68%	0.08%	1.36%	0.86%	0.75%
Spirochaetia*	0.00%	0.16%	0.70%	0.26%	0.28%
Mollicutes	0.14%	0.30%	0.48%	0.14%	0.27%
Erysipelotrichia*	0.32%	0.14%	0.16%	0.34%	0.24%
Acidimicrobiia	0.10%	0.00%	0.02%	0.12%	0.06%
Synergistia*	0.00%	0.18%	0.00%	0.06%	0.06%
Deltaproteobacteria*	0.18%	0.00%	0.00%	0.00%	0.05%
Dehalococcoidia	0.06%	0.00%	0.00%	0.00%	0.01%
Planctomycetacia*	0.06%	0.00%	0.00%	0.00%	0.01%
Deinococci	0.02%	0.00%	0.00%	0.00%	0.00%
Nitrospinia	0.02%	0.00%	0.00%	0.00%	0.00%
Coriobacteriia	0.02%	0.00%	0.00%	0.00%	0.00%
Others	2.42%	0.71%	1.96%	1.48%	1.64%

[b] Classes belonging to the core microbiome are highlighted.

* Classes have also been reported as pig tonsillar microbiota in a previous study (Lowe et al., BMC microbiology. 2012).

Complete list of all order identified

**Order**[c]	**Tonsil.1**	**Tonsil.2**	**Tonsil.3**	**Tonsil.4**	**% of total**
Bacteroidales*	39.41%	4.93%	41.43%	19.98%	26.44%
Clostridiales*	8.30%	34.05%	24.67%	23.35%	22.59%
Fusobacteriales*	11.67%	3.19%	9.00%	35.78%	14.91%
Lactobacillales*	7.54%	51.05%	2.77%	7.96%	17.33%
Pasteurellales*	13.17%	0.34%	3.15%	1.82%	4.62%

Pseudomonadales*	6.96%	0.00%	5.29%	0.56%	3.20%
Flavobacteriales*	3.73%	0.00%	3.91%	1.92%	2.39%
Selenomonadales	0.32%	4.49%	2.22%	0.28%	1.83%
Campylobacterales*	0.42%	0.72%	1.64%	1.06%	0.96%
Bacillales*	1.46%	0.00%	0.28%	0.20%	0.48%
Xanthomonadales*	0.52%	0.12%	0.38%	0.86%	0.47%
Rhodobacterales*	0.32%	0.00%	0.58%	0.38%	0.32%
Enterobacteriales*	0.16%	0.00%	0.98%	0.02%	0.29%
Spirochaetales*	0.00%	0.16%	0.70%	0.26%	0.28%
Mycoplasmatales*	0.14%	0.30%	0.48%	0.14%	0.27%
Actinomycetales*	0.78%	0.00%	0.12%	0.06%	0.24%
Erysipelotrichales*	0.32%	0.14%	0.16%	0.34%	0.24%
Sphingomonadales	0.10%	0.04%	0.40%	0.34%	0.22%
Chitinophagales	0.02%	0.00%	0.24%	0.24%	0.13%
Rhizobiales	0.18%	0.00%	0.18%	0.10%	0.12%
Oceanospirillales	0.32%	0.00%	0.00%	0.00%	0.08%
Caulobacterales*	0.08%	0.04%	0.16%	0.04%	0.08%
Sphingobacteriales*	0.02%	0.00%	0.18%	0.06%	0.07%
Synergistales*	0.00%	0.18%	0.00%	0.06%	0.06%
Nostocales	0.06%	0.00%	0.04%	0.06%	0.04%
Aeromonadales	0.04%	0.00%	0.10%	0.00%	0.04%
Cytophagales	0.04%	0.00%	0.06%	0.02%	0.03%
Cellvibrionales	0.10%	0.00%	0.00%	0.00%	0.03%
Micrococcales	0.06%	0.00%	0.04%	0.00%	0.03%
Cardiobacteriales*	0.00%	0.06%	0.00%	0.00%	0.01%
Bradymonadales	0.04%	0.00%	0.00%	0.00%	0.01%
Bdellovibrionales	0.04%	0.00%	0.00%	0.00%	0.01%
Nitrospinales	0.02%	0.00%	0.00%	0.00%	0.00%
Alteromonadales	0.02%	0.00%	0.00%	0.00%	0.00%
Coriobacteriales*	0.02%	0.00%	0.00%	0.00%	0.00%
Deinococcales	0.02%	0.00%	0.00%	0.00%	0.00%
Propionibacteriales	0.02%	0.00%	0.00%	0.00%	0.00%
Rhodospirillales*	0.00%	0.00%	0.02%	0.00%	0.00%
Other	3.58%	0.19%	0.82%	4.11%	2.18%

[c] Orderes belonging to the core microbiome are highlighted.

* Orders have also been reported as pig tonsillar microbiota in a previous study (Lowe et al., BMC microbiology. 2012).

Complete list of all family identified

**Family[d]**	**Tonsil.1**	**Tonsil.2**	**Tonsil.3**	**Tonsil.4**	**% of total**
Bacteroidaceae*	34.38%	1.86%	29.24%	9.00%	18.62%
Streptococcaceae*	7.32%	51.05%	2.69%	7.88%	17.24%
Fusobacteriaceae*	11.22%	3.15%	9.00%	35.58%	14.74%
Peptostreptococcaceae*	3.11%	20.04%	14.45%	5.87%	10.87%
Porphyromonadaceae*	4.83%	1.64%	11.81%	10.02%	7.08%
Lachnospiraceae*	0.70%	4.19%	1.04%	13.09%	4.76%
Pasteurellaceae*	13.17%	0.34%	3.15%	1.82%	4.62%
Moraxellaceae*	6.86%	0.00%	5.23%	0.54%	3.16%
Veillonellaceae*	0.32%	4.49%	2.20%	0.24%	1.81%
Defluviitaleaceae	4.07%	0.12%	0.94%	0.30%	1.36%
Weeksellaceae	1.40%	0.00%	2.91%	0.78%	1.27%
Campylobacteraceae*	0.42%	0.72%	1.64%	1.06%	0.96%
Neisseriaceae*	1.08%	0.14%	0.14%	2.27%	0.91%
Burkholderiaceae*	0.96%	0.06%	0.46%	1.44%	0.73%
Prevotellaceae*	0.12%	1.28%	0.24%	0.82%	0.62%
Flavobacteriaceae*	1.04%	0.00%	0.52%	0.58%	0.54%
Xanthomonadaceae*	0.46%	0.12%	0.38%	0.86%	0.46%

Staphylococcaceae*	1.46%	0.00%	0.04%	0.16%	0.42%
Rhodobacteraceae*	0.32%	0.00%	0.58%	0.38%	0.32%
Enterobacteriaceae*	0.16%	0.00%	0.98%	0.02%	0.29%
Spirochaetaceae*	0.00%	0.16%	0.70%	0.26%	0.28%
Mycoplasmataceae*	0.14%	0.30%	0.48%	0.14%	0.27%
Actinomycetaceae*	0.78%	0.00%	0.12%	0.06%	0.24%
Erysipelotrichaceae*	0.32%	0.14%	0.16%	0.34%	0.24%
Sphingomonadaceae	0.10%	0.04%	0.40%	0.34%	0.22%
Leptotrichiaceae*	0.46%	0.04%	0.00%	0.20%	0.18%
Chitinophagaceae*	0.02%	0.00%	0.24%	0.24%	0.13%
Ruminococcaceae*	0.08%	0.00%	0.00%	0.36%	0.11%
Rhizobiaceae	0.12%	0.00%	0.14%	0.08%	0.08%
Caulobacteraceae*	0.08%	0.04%	0.16%	0.04%	0.08%
Sphingobacteriaceae	0.02%	0.00%	0.18%	0.06%	0.06%
Aerococcaceae*	0.18%	0.00%	0.00%	0.06%	0.06%
Synergistaceae*	0.00%	0.18%	0.00%	0.06%	0.06%
Paludibacteraceae	0.00%	0.02%	0.12%	0.04%	0.05%
Pseudomonadaceae*	0.10%	0.00%	0.06%	0.02%	0.05%
Halomonadaceae	0.18%	0.00%	0.00%	0.00%	0.05%
Listeriaceae	0.00%	0.00%	0.18%	0.00%	0.05%
Aeromonadaceae	0.04%	0.00%	0.10%	0.00%	0.04%
Tannerellaceae	0.00%	0.08%	0.02%	0.04%	0.04%
Alcanivoracaceae	0.14%	0.00%	0.00%	0.00%	0.04%
Carnobacteriaceae*	0.04%	0.00%	0.06%	0.02%	0.03%
Xenococcaceae	0.04%	0.00%	0.02%	0.04%	0.02%
Planococcaceae	0.00%	0.00%	0.06%	0.04%	0.02%
Spongiibacteraceae	0.10%	0.00%	0.00%	0.00%	0.02%
Christensenellaceae	0.00%	0.04%	0.00%	0.04%	0.02%
Spirosomaceae	0.00%	0.00%	0.06%	0.02%	0.02%
Intrasporangiaceae	0.06%	0.00%	0.02%	0.00%	0.02%
Acidaminococcaceae	0.00%	0.00%	0.02%	0.04%	0.01%
Cardiobacteriaceae*	0.00%	0.06%	0.00%	0.00%	0.01%
Chromobacteriaceae	0.00%	0.00%	0.06%	0.00%	0.01%
Rhodanobacteraceae	0.06%	0.00%	0.00%	0.00%	0.01%
Cyclobacteriaceae	0.04%	0.00%	0.00%	0.00%	0.01%
Bacteriovoracaceae	0.04%	0.00%	0.00%	0.00%	0.01%
Peptococcaceae	0.00%	0.00%	0.00%	0.04%	0.01%
Atopobiaceae	0.02%	0.00%	0.00%	0.00%	0.00%
Dermatophilaceae	0.00%	0.00%	0.02%	0.00%	0.00%
Beijerinckiaceae	0.02%	0.00%	0.00%	0.00%	0.00%
Nitrospinaceae	0.02%	0.00%	0.00%	0.00%	0.00%
Rhodospirillaceae	0.00%	0.00%	0.02%	0.00%	0.00%
Deinococcaceae	0.02%	0.00%	0.00%	0.00%	0.00%
Propionibacteriaceae	0.02%	0.00%	0.00%	0.00%	0.00%
Alteromonadaceae	0.02%	0.00%	0.00%	0.00%	0.00%
Leuconostocaceae	0.00%	0.00%	0.02%	0.00%	0.00%
Other	3.34%	9.70%	8.94%	4.71%	6.67%

[d] Families belonging to the core microbiome are highlighted.

* Families have also been reported as pig tonsillar microbiota in a previous study (Lowe et al., BMC microbiology. 2012).

Complete list of all genus identified

**Genus[e]**	**Tonsil.1**	**Tonsil.2**	**Tonsil.3**	**Tonsil.4**	**% of total**
Bacteroides*	34.38%	1.86%	29.24%	9.00%	18.62%
Fusobacterium*	11.22%	3.15%	9.00%	35.58%	14.74%
Streptococcus*	5.07%	41.79%	2.47%	7.20%	14.13%
Helcococcus*	2.33%	9.76%	13.67%	4.79%	7.64%

Porphyromonas*	4.83%	1.64%	11.81%	10.02%	7.08%
Gemella*	2.24%	9.26%	0.22%	0.68%	3.10%
Pasteurella*	8.02%	0.00%	2.93%	0.04%	2.75%
Peptostreptococcus*	0.04%	9.82%	0.58%	0.36%	2.70%
Acetitomaculum	0.04%	0.02%	0.02%	8.74%	2.21%
Actinobacillus*	5.15%	0.34%	0.22%	1.78%	1.87%
Acinetobacter*	1.64%	0.00%	4.87%	0.18%	1.67%
Moraxella*	5.03%	0.00%	0.04%	0.20%	1.32%
Chryseobacterium	1.40%	0.00%	2.91%	0.78%	1.27%
Veillonella*	0.02%	4.09%	0.60%	0.08%	1.20%
Campylobacter*	0.42%	0.72%	1.64%	1.06%	0.96%
Flavobacterium	1.00%	0.00%	0.46%	0.52%	0.50%
Parvimonas*	0.02%	0.30%	0.74%	0.72%	0.45%
Staphylococcus*	1.46%	0.00%	0.04%	0.16%	0.42%
Bergeyella	0.98%	0.00%	0.00%	0.28%	0.32%
Mycoplasma*	0.14%	0.30%	0.48%	0.14%	0.27%
Acidovorax*	0.28%	0.02%	0.26%	0.40%	0.24%
Paracoccus*	0.16%	0.00%	0.46%	0.32%	0.24%
Proteocatella*	0.10%	0.28%	0.10%	0.40%	0.22%
Soonwooa	0.26%	0.00%	0.34%	0.28%	0.22%
Trueperella	0.62%	0.00%	0.04%	0.04%	0.18%
Leptotrichia*	0.46%	0.04%	0.00%	0.16%	0.17%
Thermomonas	0.20%	0.06%	0.10%	0.30%	0.17%
Rhizorhapis	0.08%	0.00%	0.26%	0.22%	0.14%
Luteimonas	0.02%	0.06%	0.14%	0.30%	0.13%
Alloprevotella	0.06%	0.04%	0.16%	0.20%	0.12%
Ralstonia	0.04%	0.00%	0.00%	0.40%	0.11%
Psychrobacter*	0.10%	0.00%	0.18%	0.10%	0.10%
Filifactor*	0.14%	0.10%	0.02%	0.10%	0.09%
Rhodobacter	0.16%	0.00%	0.12%	0.06%	0.08%
Brevundimonas*	0.08%	0.04%	0.16%	0.04%	0.08%
Prevotella*	0.00%	0.16%	0.06%	0.10%	0.08%
Filobacterium	0.00%	0.00%	0.10%	0.20%	0.08%
Enhydrobacter	0.10%	0.00%	0.14%	0.06%	0.08%
Actinomyces*	0.16%	0.00%	0.08%	0.02%	0.07%
Pseudoxanthomonas	0.08%	0.00%	0.04%	0.12%	0.06%
Catonella	0.00%	0.00%	0.20%	0.00%	0.05%
Fretibacterium	0.00%	0.18%	0.00%	0.02%	0.05%
Alcaligenes	0.16%	0.00%	0.00%	0.02%	0.05%
Comamonas	0.02%	0.00%	0.04%	0.12%	0.05%
Faecalibacterium	0.06%	0.00%	0.00%	0.12%	0.05%
Stenotrophobacter	0.10%	0.00%	0.04%	0.04%	0.05%
Pseudomonas*	0.10%	0.00%	0.06%	0.02%	0.05%
Peptoniphilus*	0.04%	0.00%	0.08%	0.06%	0.05%
Halomonas	0.18%	0.00%	0.00%	0.00%	0.05%
Brochothrix	0.00%	0.00%	0.18%	0.00%	0.05%
Globicatella	0.14%	0.00%	0.00%	0.04%	0.05%
Capnocytophaga*	0.04%	0.00%	0.06%	0.06%	0.04%
Pelistega	0.14%	0.00%	0.00%	0.02%	0.04%
Aeromonas	0.04%	0.00%	0.10%	0.00%	0.04%
Tannerella*	0.00%	0.08%	0.02%	0.04%	0.04%
Alcanivorax	0.14%	0.00%	0.00%	0.00%	0.04%
Proteus*	0.00%	0.00%	0.12%	0.00%	0.03%
Stenotrophomonas	0.12%	0.00%	0.00%	0.00%	0.03%
Sphingobacterium	0.00%	0.00%	0.10%	0.02%	0.03%
Niabella	0.02%	0.00%	0.08%	0.02%	0.03%
Blastocatella	0.06%	0.00%	0.02%	0.04%	0.03%
Mitsuaria	0.02%	0.00%	0.04%	0.04%	0.03%

Alicycliphilus	0.00%	0.00%	0.00%	0.10%	0.03%
Spongiibacter	0.10%	0.00%	0.00%	0.00%	0.03%
Delftia	0.08%	0.00%	0.00%	0.02%	0.03%
Fastidiosipila	0.02%	0.00%	0.00%	0.08%	0.03%
Lysinibacillus	0.00%	0.00%	0.04%	0.04%	0.02%
Brachymonas	0.02%	0.00%	0.02%	0.04%	0.02%
Shinella	0.04%	0.00%	0.00%	0.04%	0.02%
Diaphorobacter	0.04%	0.00%	0.00%	0.04%	0.02%
Moheibacter	0.02%	0.00%	0.06%	0.00%	0.02%
Johnsonella	0.00%	0.02%	0.00%	0.06%	0.02%
Tetrasphaera	0.06%	0.00%	0.02%	0.00%	0.02%
Hydrogenophaga	0.02%	0.00%	0.00%	0.06%	0.02%
Arenimonas	0.02%	0.00%	0.06%	0.00%	0.02%
Mesorhizobium	0.00%	0.00%	0.06%	0.02%	0.02%
Ottowia	0.08%	0.00%	0.00%	0.00%	0.02%
Eubacterium*	0.00%	0.00%	0.02%	0.04%	0.01%
Blastomonas	0.00%	0.04%	0.02%	0.00%	0.01%
Succiniclasticum	0.00%	0.00%	0.02%	0.04%	0.01%
Granulicatella	0.04%	0.00%	0.00%	0.02%	0.01%
Aquabacterium	0.00%	0.02%	0.02%	0.02%	0.01%
Qipengyuania	0.02%	0.00%	0.02%	0.02%	0.01%
Fusibacter	0.06%	0.00%	0.00%	0.00%	0.01%
Lachnoanaerobaculum	0.00%	0.00%	0.00%	0.06%	0.01%
Neisseria*	0.00%	0.00%	0.00%	0.06%	0.01%
Suttonella*	0.00%	0.06%	0.00%	0.00%	0.01%
Proteiniphilum	0.00%	0.06%	0.00%	0.00%	0.01%
Klebsiella	0.00%	0.00%	0.02%	0.02%	0.01%
Terrimonas*	0.00%	0.00%	0.04%	0.00%	0.01%
Roseivirga	0.04%	0.00%	0.00%	0.00%	0.01%
Pyramidobacter*	0.00%	0.00%	0.00%	0.04%	0.01%
Finegoldia*	0.04%	0.00%	0.00%	0.00%	0.01%
Streptobacillus	0.00%	0.00%	0.00%	0.04%	0.01%
Peptococcus*	0.00%	0.00%	0.00%	0.04%	0.01%
Peredibacter	0.00%	0.00%	0.00%	0.00%	0.01%
Sphingorhabdus	0.00%	0.00%	0.02%	0.02%	0.01%
Silanimonas	0.00%	0.00%	0.02%	0.02%	0.01%
Kurthia	0.00%	0.00%	0.02%	0.00%	0.00%
Lactococcus	0.02%	0.00%	0.00%	0.00%	0.00%
Methylobacterium*	0.02%	0.00%	0.00%	0.00%	0.00%
Leuconostoc	0.00%	0.00%	0.02%	0.00%	0.00%
Lysobacter	0.00%	0.00%	0.02%	0.00%	0.00%
Leadbetterella	0.00%	0.00%	0.00%	0.02%	0.00%
Porphyrobacter	0.00%	0.00%	0.00%	0.02%	0.00%
Sphingopyxis	0.00%	0.00%	0.00%	0.02%	0.00%
Paludibacterium*	0.00%	0.00%	0.02%	0.00%	0.00%
Devosia	0.00%	0.00%	0.02%	0.00%	0.00%
Runella	0.00%	0.00%	0.02%	0.00%	0.00%
Cloacibacterium	0.00%	0.00%	0.02%	0.00%	0.00%
Alteromonas	0.02%	0.00%	0.00%	0.00%	0.00%
Haematospirillum	0.00%	0.00%	0.02%	0.00%	0.00%
Nitrospina	0.02%	0.00%	0.00%	0.00%	0.00%
Ferruginibacter	0.00%	0.00%	0.02%	0.00%	0.00%
Deinococcus	0.02%	0.00%	0.00%	0.00%	0.00%
Carnobacterium	0.00%	0.00%	0.02%	0.00%	0.00%
Gulbenkiania	0.00%	0.00%	0.02%	0.00%	0.00%
Dokdonella	0.02%	0.00%	0.00%	0.00%	0.00%
Romboutsia	0.00%	0.00%	0.00%	0.02%	0.00%
Cutibacterium	0.02%	0.00%	0.00%	0.00%	0.00%

Achromobacter	0.00%	0.00%	0.02%	0.00%	0.00%
Atopobium*	0.02%	0.00%	0.00%	0.00%	0.00%
Other	9.19%	15.69%	13.46%	12.17%	12.64%

[e] Genera belonging to the core microbiome are highlighted.

* Genera have also been reported as pig tonsillar microbiota in a previous study (Lowe et al., BMC microbiology. 2012).

Complete list of all Species identified

**Species[f]**	**Tonsil.1**	**Tonsil.2**	**Tonsil.3**	**Tonsil.4**	**% of total**
Streptococcus_suis	4.39%	41.73%	1.78%	6.17%	13.52%
Fusobacterium_sp	8.30%	1.92%	4.37%	5.89%	5.12%
Prevotella_heparinolytica	14.37%	0.86%	3.09%	0.36%	4.67%
Gemella_palaticanis	2.24%	9.26%	0.22%	0.68%	3.10%
Lachnospiraceae_bacterium_canine_oral_taxon_099	0.04%	0.02%	0.02%	8.74%	2.21%
Porphyromonas_macacae	0.06%	0.00%	4.11%	0.16%	1.08%
Campylobacter_mucosalis	0.42%	0.72%	1.56%	1.02%	0.93%
Acinetobacter_lwoffii	0.44%	0.00%	2.67%	0.02%	0.78%
[Haemophilus]_parasuis	0.68%	0.28%	0.04%	1.68%	0.67%
Selenomonas_sp_oral_clone_JI021	0.30%	0.40%	1.60%	0.16%	0.62%
[Eubacterium]_minutum	0.00%	1.60%	0.04%	0.04%	0.42%
Porphyromonas_sp_oral_clone_P4GB_100_P2	0.72%	0.06%	0.34%	0.48%	0.40%
[Eubacterium]_brachy	0.00%	0.00%	0.04%	1.56%	0.40%
Staphylococcus_aureus	1.18%	0.00%	0.00%	0.08%	0.32%
Capnocytophaga_sp_canine_oral_taxon_339	0.76%	0.00%	0.00%	0.46%	0.31%
Escherichia_coli	0.16%	0.00%	0.84%	0.00%	0.25%
Peptostreptococcaceae_bacterium_canine_oral_taxon_333	0.04%	0.00%	0.86%	0.10%	0.25%
Soonwooa_buanensis	0.26%	0.00%	0.34%	0.28%	0.22%
Trueperella_pyogenes	0.62%	0.00%	0.04%	0.04%	0.18%
Treponema_sp_G179	0.00%	0.04%	0.46%	0.20%	0.18%
Mycoplasma_hyorhinis	0.02%	0.30%	0.36%	0.00%	0.17%
Bacteroides_pyogenes	0.40%	0.06%	0.00%	0.16%	0.16%
Acinetobacter_schindleri	0.10%	0.00%	0.40%	0.00%	0.13%
Peptostreptococcaceae_bacterium_canine_oral_taxon_221	0.00%	0.06%	0.22%	0.10%	0.10%
Streptococcus_porcinus	0.00%	0.00%	0.08%	0.28%	0.09%
Chryseobacterium_haifense	0.10%	0.00%	0.22%	0.04%	0.09%
Psychrobacter_sp_PRwf-1	0.06%	0.00%	0.16%	0.10%	0.08%
Filobacterium_rodentium	0.00%	0.00%	0.10%	0.20%	0.08%
Paracoccus_sp_J6	0.04%	0.00%	0.12%	0.12%	0.07%
Brevundimonas_nasdae	0.08%	0.04%	0.12%	0.02%	0.07%
Actinomyces_hyovaginalis	0.16%	0.00%	0.08%	0.02%	0.07%
Porphyromonas_loveana	0.00%	0.04%	0.16%	0.04%	0.06%
Acinetobacter_baumannii	0.06%	0.00%	0.14%	0.04%	0.06%
Porphyromonas_crevioricanis	0.06%	0.00%	0.14%	0.04%	0.06%
Filifactor_villosus	0.06%	0.08%	0.02%	0.06%	0.06%
Synergistales_bacterium_canine_oral_taxon_244	0.00%	0.18%	0.00%	0.02%	0.05%
Porphyromonadaceae_bacterium_COT-184_OH4590	0.00%	0.02%	0.12%	0.04%	0.05%
Alcaligenes_faecalis	0.16%	0.00%	0.00%	0.02%	0.05%
Comamonas_testosteroni	0.02%	0.00%	0.04%	0.12%	0.05%
Chryseobacterium_hominis	0.02%	0.00%	0.14%	0.02%	0.05%
Treponema_sp_canine_oral_taxon_35[illegible]	0.00%	0.12%	0.02%	0.02%	0.04%

Bacteroides_fragilis	0.06%	0.04%	0.04%	0.02%	0.04%
Aeromonas_veronii	0.04%	0.00%	0.10%	0.00%	0.04%
Tannerella_forsythia	0.00%	0.08%	0.02%	0.04%	0.04%
Frigovirgula_sp_canine_oral_taxon_05	0.02%	0.06%	0.02%	0.04%	0.04%
Streptococcus_sp_Str-122	0.00%	0.00%	0.00%	0.14%	0.04%
Proteus_mirabilis	0.00%	0.00%	0.12%	0.00%	0.03%
Stenotrophomonas_maltophilia	0.12%	0.00%	0.00%	0.00%	0.03%
Erysipelotrichaceae_bacterium_canine_oral_taxon_255	0.04%	0.04%	0.00%	0.04%	0.03%
Porphyromonas_cangingivalis	0.02%	0.00%	0.10%	0.00%	0.03%
Prevotella_sp_canine_oral_taxon_282	0.00%	0.04%	0.00%	0.08%	0.03%
TM7_phylum_sp_oral_clone_FR058	0.06%	0.00%	0.00%	0.06%	0.03%
Mitsuaria_chitosanitabida	0.02%	0.00%	0.04%	0.04%	0.02%
Halomonas_meridiana	0.10%	0.00%	0.00%	0.00%	0.02%
Mycoplasma_hyosynoviae	0.00%	0.00%	0.10%	0.00%	0.02%
Mycoplasma_lipophilum	0.08%	0.00%	0.00%	0.02%	0.02%
Clostridiales_bacterium_canine_oral_taxon_216	0.02%	0.00%	0.00%	0.08%	0.02%
Pseudomonas_fragi	0.08%	0.00%	0.02%	0.00%	0.02%
Delftia_tsuruhatensis	0.08%	0.00%	0.00%	0.02%	0.02%
Lysinibacillus_sp_FFL6	0.00%	0.00%	0.04%	0.04%	0.02%
Shinella_zoogloeoides	0.04%	0.00%	0.00%	0.04%	0.02%
Pseudomonas_aeruginosa	0.02%	0.00%	0.04%	0.02%	0.02%
Clostridium_perfringens	0.08%	0.00%	0.00%	0.00%	0.02%
Brevundimonas_terrae	0.00%	0.00%	0.04%	0.02%	0.01%
Chryseobacterium_indologenes	0.02%	0.00%	0.02%	0.02%	0.01%
Hydrocoleum_sp_SAG_3887	0.02%	0.00%	0.02%	0.02%	0.01%
Peptostreptococcaceae_bacterium_canine_oral_taxon_019	0.06%	0.00%	0.00%	0.00%	0.01%
Clostridiales_bacterium_canine_oral_taxon_100	0.00%	0.00%	0.00%	0.06%	0.01%
Suttonella_ornithocola	0.00%	0.06%	0.00%	0.00%	0.01%
Klebsiella_pneumoniae	0.00%	0.00%	0.02%	0.02%	0.01%
Roseivirga_spongicola	0.04%	0.00%	0.00%	0.00%	0.01%
Deltaproteobacteria_bacterium_CSP1-	0.04%	0.00%	0.00%	0.00%	0.01%
[Eubacterium]_infirmum	0.00%	0.00%	0.00%	0.04%	0.01%
Capnocytophaga_sp_canine_oral_taxon_329	0.00%	0.00%	0.00%	0.00%	0.01%
Mycoplasma_flocculare	0.04%	0.00%	0.00%	0.00%	0.01%
Flavobacterium_hauense	0.04%	0.00%	0.00%	0.00%	0.01%
bacterium_BYJ5-1	0.00%	0.00%	0.04%	0.00%	0.01%
Peptostreptococcaceae_bacterium_feline_oral_taxon_137	0.00%	0.00%	0.00%	0.04%	0.01%
Peptostreptococcaceae_bacterium_canine_oral_taxon_332	0.00%	0.00%	0.00%	0.04%	0.01%
alpha_proteobacterium_A0839	0.00%	0.00%	0.02%	0.02%	0.01%
Lachnospiraceae_bacterium_feline_oral_taxon_008	0.02%	0.00%	0.00%	0.00%	0.00%
Haematospirillum_jordaniae	0.00%	0.00%	0.02%	0.00%	0.00%
Deinococcus_grandis	0.02%	0.00%	0.00%	0.00%	0.00%
beta_proteobacterium_on7	0.00%	0.00%	0.02%	0.00%	0.00%
Moraxella_bovoculi	0.02%	0.00%	0.00%	0.00%	0.00%
Sphingobacterium_alimentarium	0.00%	0.00%	0.02%	0.00%	0.00%
candidate_division_TM7_bacterium_JGI_0001002-L20	0.02%	0.00%	0.00%	0.00%	0.00%
Alphaproteobacteria_bacterium_CG1_02_46_17	0.00%	0.00%	0.02%	0.00%	0.00%
Acinetobacter_gandensis	0.00%	0.00%	0.02%	0.00%	0.00%

Hyaloperonospora_arabidopsidis	0.00%	0.00%	0.00%	0.02%	0.00%
Sphingobacterium_multivorum	0.00%	0.00%	0.02%	0.00%	0.00%
Others	62.46%	41.89%	74.02%	69.44%	61.96%

[f] Species belonging to the core microbiome are highlighted.

Table S7. The core microbiome of porcine tonsils

Phylum	% of total	Class	% of total	Order	% of total	Family	% of total	Genus	% of total
Firmicutes	42.47	*Clostridia*	22.59	*Clostridiales*	22.59	*Peptostreptococcaceae*	10.87	*Helcococcus*	7.64
								Peptostreptococcus	2.70
						Lachnospiraceae	4.76	*Acetitomaculum*	2.21
						Defluviitaleaceae	1.36		
		Bacilli	17.82	*Lactobacillales*	17.33	*Streptococcaceae*	17.24	*Streptococcus*	14.13
								Gemella	3.10
		Negativicutes	1.83	*Selenomonadales*	1.83	*Veillonellaceae*	1.81	*Veillonella*	1.20
Proteobacteria	11.23	*Gammaproteobacteria*	10.43	*Pasteurellales*	4.62	*Pasteurellaceae*	4.62	*Pasteurella*	2.75
								Actinobacillus	1.87
				Pseudomonadales	3.20	*Moraxellaceae*	3.16	*Acinetobacter*	1.67
								Moraxella	1.32
Bacteroidetes	29.05	*Bacteroidia*	29.05	*Bacteroidales*	26.44	*Bacteroidaceae*	18.62	*Bacteroides*	18.62
						Porphyromonadaceae	7.08	*Porphyromonas*	7.08
				Flavobacteriales	2.39	*Weeksellaceae*	1.27	*Chryseobacterium*	1.27
Fusobacteria	14.91	*Fusobacteriia*	14.91	*Fusobacteriales*	14.91	*Fusobacteriaceae*	14.74	*Fusobacterium*	14.74
4/15 phyla identified	97.66	6/18 class identified	96.63	8/38 order identified	93.31	11/63 family identified	85.53	14/122 genus identified	80.30

Table S8. Complete list of all taxon identified in culture-dependent microbiome (control group)

Complete list of all phyla identified

Phylum[a]	Tonsil.1	Tonsil.2	Tonsil.3	Tonsil.4	% of total
Proteobacteria	51.75%	78.79%	57.33%	74.60%	65.62%
Firmicutes	48.25%	21.21%	42.61%	25.40%	34.37%
Bacteroidetes	0.00%	0.00%	0.04%	0.00%	0.01%
Others	0.00%	0.00%	0.02%	0.00%	0.00%

[a] Phyla that comprise the core microbiome are highlighted

Complete list of all class identified

Class[b]	Tonsil.1	Tonsil.2	Tonsil.3	Tonsil.4	% of total
Gammaproteobacteria	51.67%	78.79%	57.33%	74.60%	65.60%
Bacilli	48.17%	21.19%	42.61%	25.40%	34.34%
Clostridia	0.08%	0.02%	0.00%	0.00%	0.03%
Deltaproteobacteria	0.06%	0.00%	0.00%	0.00%	0.02%
Bacteroidia	0.00%	0.00%	0.04%	0.00%	0.01%
Alphaproteobacteria	0.02%	0.00%	0.00%	0.00%	0.00%
Others	0.00%	0.00%	0.02%	0.00%	0.00%

[b] Class that comprise the core microbiome are highlighted

Complete list of all order identified

Order[c]	Tonsil.1	Tonsil.2	Tonsil.3	Tonsil.4	% of total
Enterobacteriales	51.43%	78.75%	56.90%	73.70%	65.20%
Lactobacillales	32.07%	20.42%	34.82%	7.34%	23.66%
Bacillales	16.10%	0.78%	7.78%	18.06%	10.68%
Pseudomonadales	0.14%	0.03%	0.10%	0.88%	0.29%
Aeromonadales	0.10%	0.00%	0.32%	0.02%	0.11%
Clostridiales	0.08%	0.02%	0.00%	0.00%	0.03%
Cytophagales	0.00%	0.00%	0.04%	0.00%	0.01%
Oceanospirillales	0.00%	0.00%	0.02%	0.00%	0.00%
Caulobacterales	0.02%	0.00%	0.00%	0.00%	0.00%
Others	0.06%	0.00%	0.02%	0.00%	0.02%

[c] Order that comprise the core microbiome are highlighted

Complete list of all family identified

Family[d]	Tonsil.1	Tonsil.2	Tonsil.3	Tonsil.4	% of total
Enterobacteriaceae	51.43%	78.75%	56.89%	73.70%	65.19%
Streptococcaceae	30.87%	20.42%	34.60%	7.06%	23.24%
Staphylococcaceae	15.31%	0.78%	6.37%	14.07%	9.13%
Planococcaceae	0.80%	0.00%	0.92%	3.83%	1.39%
Enterococcaceae	1.20%	0.00%	0.22%	0.28%	0.43%
Moraxellaceae	0.14%	0.04%	0.10%	0.88%	0.29%
Aeromonadaceae	0.10%	0.00%	0.32%	0.02%	0.11%
Bacillaceae	0.00%	0.00%	0.00%	0.10%	0.03%
Cyclobacteriaceae	0.00%	0.00%	0.04%	0.00%	0.01%
Caulobacteraceae	0.02%	0.00%	0.00%	0.00%	0.00%
Halomonadaceae	0.00%	0.00%	0.02%	0.00%	0.00%
Others	0.13%	0.01%	0.52%	0.06%	0.18%

[d] Family that comprise the core microbiome are highlighted

Complete list of all genus identified

Genus[e]	Tonsil.1	Tonsil.2	Tonsil.3	Tonsil.4	% of total
Escherichia	48.93%	78.67%	54.96%	70.31%	63.22%
Streptococcus	30.25%	20.42%	34.58%	7.06%	23.08%

Macrococcus	6.79%	0.28%	6.25%	13.71%	6.76%
Staphylococcus	8.52%	0.50%	0.12%	0.34%	2.37%
Klebsiella	2.43%	0.00%	1.06%	2.59%	1.52%
Kurthia	0.52%	0.00%	0.82%	3.73%	1.27%
Enterococcus	1.10%	0.00%	0.14%	0.16%	0.35%
Acinetobacter	0.14%	0.04%	0.08%	0.88%	0.29%
Proteus	0.02%	0.00%	0.68%	0.42%	0.28%
Lactococcus	0.62%	0.00%	0.02%	0.00%	0.16%
Exiguobacterium	0.00%	0.00%	0.50%	0.06%	0.14%
Lysinibacillus	0.28%	0.00%	0.10%	0.10%	0.12%
Aeromonas	0.10%	0.00%	0.32%	0.02%	0.11%
Vagococcus	0.10%	0.00%	0.08%	0.12%	0.08%
Bacillus	0.00%	0.00%	0.00%	0.10%	0.03%
Raoultella	0.00%	0.00%	0.00%	0.06%	0.02%
Roseivirga	0.00%	0.00%	0.03%	0.00%	0.00%
Providencia	0.03%	0.00%	0.00%	0.00%	0.00%
Psychrobacter	0.00%	0.00%	0.02%	0.00%	0.00%
Brevundimonas	0.02%	0.00%	0.00%	0.00%	0.00%
Halomonas	0.00%	0.00%	0.02%	0.00%	0.00%
Others	0.15%	0.09%	0.22%	0.34%	0.20%

[e] Genus that comprise the core microbiome are highlighted

Complete list of all Species identified

Species[f]	Tonsil.1	Tonsil.2	Tonsil.3	Tonsil.4	% of total
Escherichia_coli	48.39%	78.67%	54.96%	70.31%	63.08%
Streptococcus_suis	17.74%	19.46%	29.77%	5.61%	18.15%
Streptococcus_porcinus	6.33%	0.66%	0.18%	0.30%	1.87%
Klebsiella_pneumoniae	2.43%	0.00%	1.06%	2.59%	1.52%
Kurthia_gibsonii	0.52%	0.00%	0.82%	3.69%	1.26%
Proteus_mirabilis	0.02%	0.00%	0.66%	0.42%	0.28%
Enterococcus_casseliflavus	1.02%	0.00%	0.02%	0.00%	0.26%
Streptococcus_gallolyticus_subsp_pasteurianus	0.00%	0.00%	0.02%	0.58%	0.15%
Exiguobacterium_sp_AT1b	0.00%	0.00%	0.50%	0.06%	0.14%
Staphylococcus_aureus	0.20%	0.08%	0.00%	0.26%	0.14%
Lysinibacillus_sp_FFL6	0.28%	0.00%	0.10%	0.10%	0.12%
Aeromonas_veronii	0.10%	0.00%	0.32%	0.02%	0.11%
Acinetobacter_baumannii	0.00%	0.00%	0.06%	0.22%	0.07%
Enterococcus_faecalis	0.08%	0.00%	0.02%	0.16%	0.07%
Enterococcus_hirae	0.00%	0.00%	0.10%	0.00%	0.03%
Macrococcus_caseolyticus	0.00%	0.00%	0.08%	0.02%	0.03%
Staphylococcus_sciuri	0.00%	0.00%	0.08%	0.00%	0.02%
Acinetobacter_lwoffii	0.03%	0.00%	0.00%	0.00%	0.00%
Roseivirga_spongicola	0.00%	0.00%	0.03%	0.00%	0.00%
Psychrobacter_sp_PRwf-1	0.00%	0.00%	0.02%	0.00%	0.00%
Brevundimonas_nasdae	0.02%	0.00%	0.00%	0.00%	0.00%
Halomonas_meridiana	0.00%	0.00%	0.02%	0.00%	0.00%
Others	22.84%	1.13%	11.18%	15.66%	12.70%

[f] Species that comprise the core microbiome are highlighted

Table S9. Complete list of all taxon identified in culture-dependent microbiome (Lcn351 group)

Complete list of all phyla identified

**Phylum[a]**	**Tonsil.1**	**Tonsil.2**	**Tonsil.3**	**Tonsil.4**	**% of total**
Proteobacteria	79.84%	87.19%	65.06%	66.31%	74.60%
Firmicutes	20.16%	12.81%	34.94%	33.63%	25.39%
Others	0.00%	0.00%	0.00%	0.06%	0.01%

[a] Phyla that comprise the core microbiome are highlighted

Complete list of all class identified

**Class[b]**	**Tonsil.1**	**Tonsil.2**	**Tonsil.3**	**Tonsil.4**	**% of total**
Gammaproteobacteria	79.84%	87.19%	65.06%	66.31%	74.60%
Bacilli	20.14%	12.81%	34.94%	33.63%	25.38%
Clostridia	0.02%	0.00%	0.00%	0.00%	0.00%
Others	0.00%	0.00%	0.00%	0.06%	0.02%

[b] Class that comprise the core microbiome are highlighted

Complete list of all order identified

**Order[c]**	**Tonsil.1**	**Tonsil.2**	**Tonsil.3**	**Tonsil.4**	**% of total**
Enterobacteriales	79.23%	87.13%	64.56%	66.11%	74.26%
Lactobacillales	9.64%	12.13%	28.62%	3.31%	13.43%
Bacillales	10.50%	0.68%	6.32%	30.31%	11.95%
Pseudomonadales	0.61%	0.06%	0.34%	0.20%	0.30%
Aeromonadales	0.00%	0.00%	0.16%	0.00%	0.04%
Clostridiales	0.02%	0.00%	0.00%	0.00%	0.00%
Others	0.00%	0.00%	0.00%	0.07%	0.02%

[c] Order that comprise the core microbiome are highlighted

Complete list of all family identified

**Family[d]**	**Tonsil.1**	**Tonsil.2**	**Tonsil.3**	**Tonsil.4**	**% of total**
Enterobacteriaceae	79.23%	87.13%	64.56%	66.11%	74.26%
Streptococcaceae	9.54%	12.13%	28.14%	3.29%	13.28%
Staphylococcaceae	10.06%	0.68%	5.13%	27.96%	10.96%
Bacillaceae	0.00%	0.00%	0.42%	1.60%	0.50%
Planococcaceae	0.44%	0.00%	0.77%	0.76%	0.49%
Moraxellaceae	0.62%	0.06%	0.34%	0.20%	0.30%
Enterococcaceae	0.10%	0.00%	0.48%	0.02%	0.15%
Aeromonadaceae	0.00%	0.00%	0.16%	0.00%	0.04%
Others	0.01%	0.00%	0.00%	0.06%	0.02%

[d] Family that comprise the core microbiome are highlighted

Complete list of all genus identified

**Genus[e]**	**Tonsil.1**	**Tonsil.2**	**Tonsil.3**	**Tonsil.4**	**% of total**
Escherichia	75.35%	87.13%	61.92%	64.10%	72.13%
Streptococcus	9.12%	12.13%	28.06%	3.29%	13.15%
Macrococcus	4.49%	0.16%	5.01%	27.74%	9.35%
Staphylococcus	5.43%	0.52%	0.12%	0.16%	1.56%
Klebsiella	2.99%	0.00%	1.36%	1.32%	1.42%
Exiguobacterium	0.00%	0.00%	0.42%	1.60%	0.51%
Kurthia	0.38%	0.00%	0.78%	0.72%	0.47%
Proteus	0.00%	0.00%	1.16%	0.04%	0.30%
Acinetobacter	0.60%	0.06%	0.30%	0.20%	0.29%
Enterococcus	0.10%	0.00%	0.44%	0.02%	0.14%
Lactococcus	0.42%	0.00%	0.08%	0.00%	0.13%
Aeromonas	0.00%	0.00%	0.16%	0.00%	0.04%

Plesiomonas	0.10%	0.00%	0.00%	0.00%	0.02%
Lysinibacillus	0.06%	0.00%	0.00%	0.00%	0.01%
Psychrobacter	0.02%	0.00%	0.04%	0.00%	0.01%
Vagococcus	0.00%	0.00%	0.04%	0.00%	0.01%
Providencia	0.02%	0.00%	0.00%	0.00%	0.00%
Others	0.92%	0.00%	0.11%	0.81%	0.46%

[e] Genus that comprise the core microbiome are highlighted

Complete list of all species identified

Species[f]	Tonsil.1	Tonsil.2	Tonsil.3	Tonsil.4	% of total
Escherichia_coli	74.88%	87.13%	61.92%	64.10%	72.01%
Streptococcus_suis	3.25%	11.34%	24.33%	2.35%	10.32%
Klebsiella_pneumoniae	2.99%	0.00%	1.36%	1.32%	1.42%
Streptococcus_porcinus	2.97%	0.66%	0.32%	0.06%	1.00%
Exiguobacterium_sp_AT1b	0.00%	0.00%	0.42%	1.60%	0.51%
Kurthia_gibsonii	0.38%	0.00%	0.78%	0.72%	0.47%
Proteus_mirabilis	0.00%	0.00%	1.14%	0.04%	0.30%
Staphylococcus_aureus	0.24%	0.12%	0.04%	0.10%	0.13%
Enterococcus_hirae	0.00%	0.00%	0.36%	0.00%	0.09%
Acinetobacter_baumannii	0.12%	0.02%	0.06%	0.10%	0.07%
Aeromonas_veronii	0.00%	0.00%	0.16%	0.00%	0.04%
Enterococcus_casseliflavus	0.06%	0.00%	0.04%	0.00%	0.03%
Enterococcus_faecalis	0.04%	0.00%	0.04%	0.02%	0.03%
Acinetobacter_lwoffii	0.04%	0.00%	0.02%	0.00%	0.01%
Psychrobacter_sp_PRwf-1	0.02%	0.00%	0.04%	0.00%	0.01%
Lysinibacillus_sp_FFL6	0.06%	0.00%	0.00%	0.00%	0.01%
Macrococcus_caseolyticus	0.00%	0.00%	0.00%	0.06%	0.01%
Streptococcus_salivarius_subsp_salivarius	0.00%	0.00%	0.04%	0.00%	0.01%
Streptococcus_sp_Str-122	0.00%	0.00%	0.02%	0.00%	0.00%
Others	14.95%	0.73%	8.91%	29.53%	13.53%

[f] Species that comprise the core microbiome are highlighted
