# Supplementary material for: The antimicrobial systems of Streptococcus suis promote niche competition in pig tonsils
Source: Virulence. 2022 Apr 28;13(1):781–93. doi: 10.1080/21505594.2022.2069390 (PMC9067509; doi:10.1080/21505594.2022.2069390)
Supplement: Supplemental Material [file KVIR_A_2069390_SM0560.zip › supplementary/Supplementary figures S1-S6.docx]

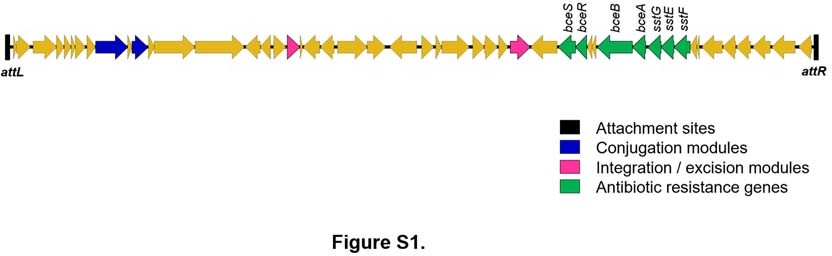


**Figure S1.** The ICE_WUSS351_ of strain WUSS351. The ICE_WUSS351_ (from *E8M06_RS04990* to *E8M06_RS05235*) contains an ATT sequence of 15 bp: 5' -TTATTTAAGAGTAAC -3' in the flanking region, which harbors the bacitracin resistance genes *sstFEG*, *bceBA*, and *bceSR*.


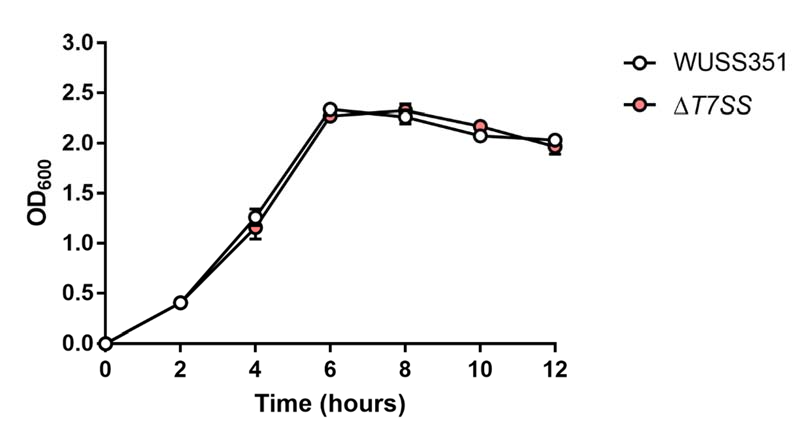


**Figure S2.** Growth curves of strains WUSS351 and Δ*T7SS* in THB medium. The results are shown as the means ± SEM (n = 3).


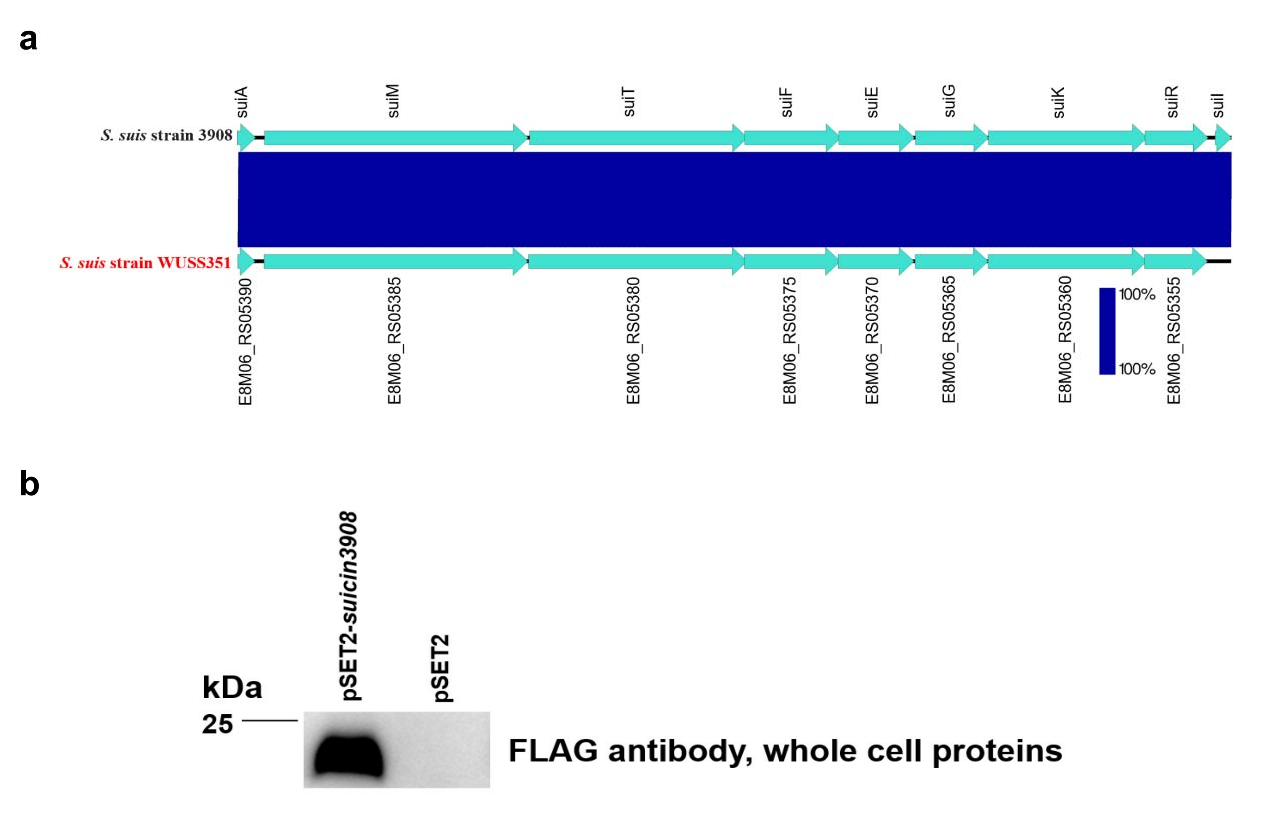


**Figure S3.** Suicin3908 of Strain WUSS351. (a) Comparison of the sequences of lantibiotics *suicin* clusters between strains WUSS351 and 3908. (b) Probing of whole-cell proteins extracted from strain WUSS351 with anti-FLAG antibodies.


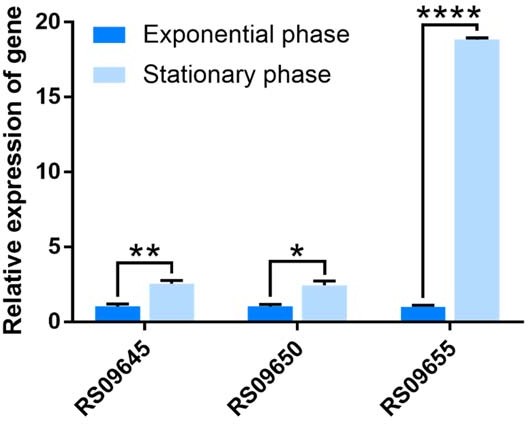


**Figure S4.** Relative mRNA expression of *Lcn351.* The relative mRNA expression levels of *RS09645*, *RS09650*, and *RS09655* in strain WUSS351 grown to the stationary phase were compared with their expression levels in the exponential phase. The graph shows the means ± SEM from three independent experiments; asterisks indicate pairs of significantly different values (n = 3, **** indicates p < 0.0001, ** indicates p < 0.01, * indicates p < 0.05, two-tailed unpaired *t* test).


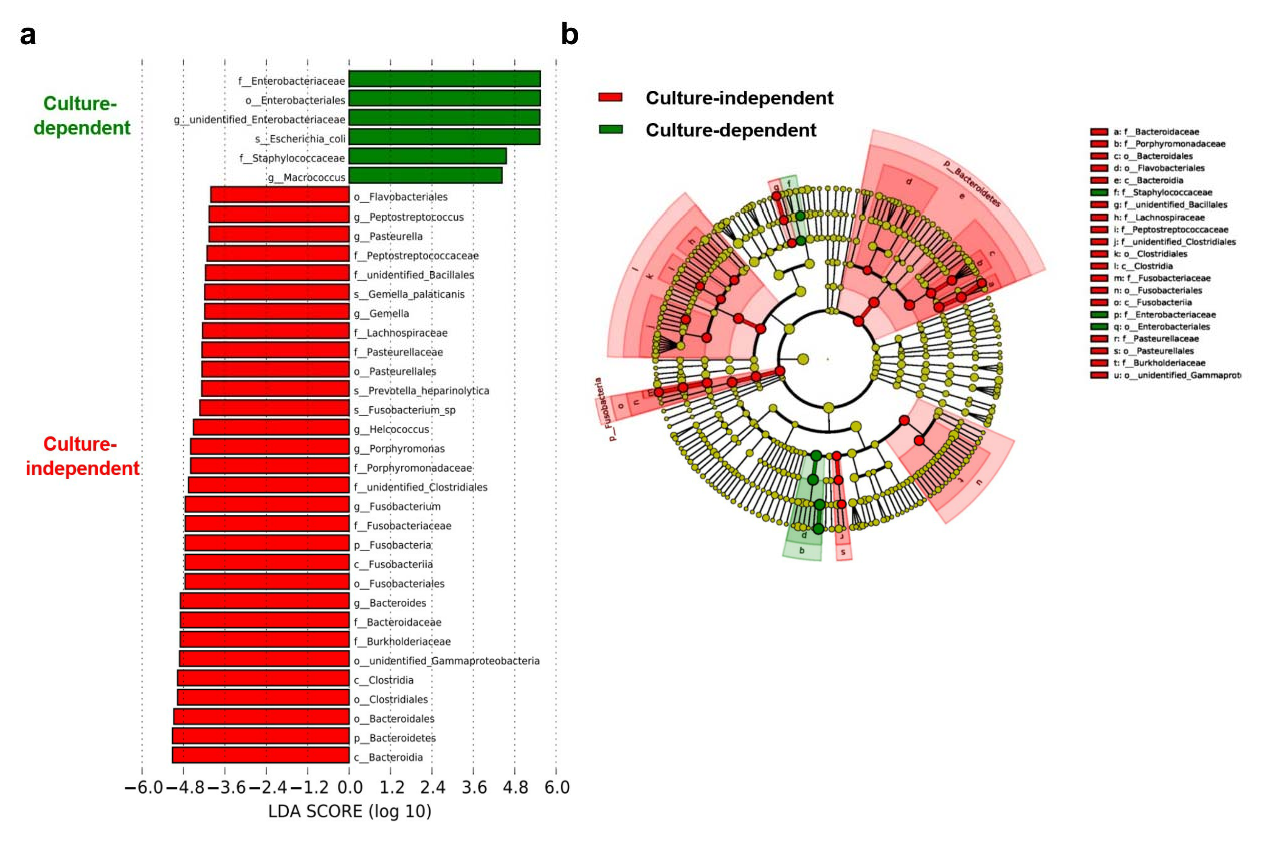


**Figure S5.** LEfSe (LDA Effect Size) analysis. (a) Histogram of the LDA scores and (b) cladogram computed for features that were differentially abundant between the microbiota of culture‑independent (red) and culture-dependent (in the control group) (green) groups.


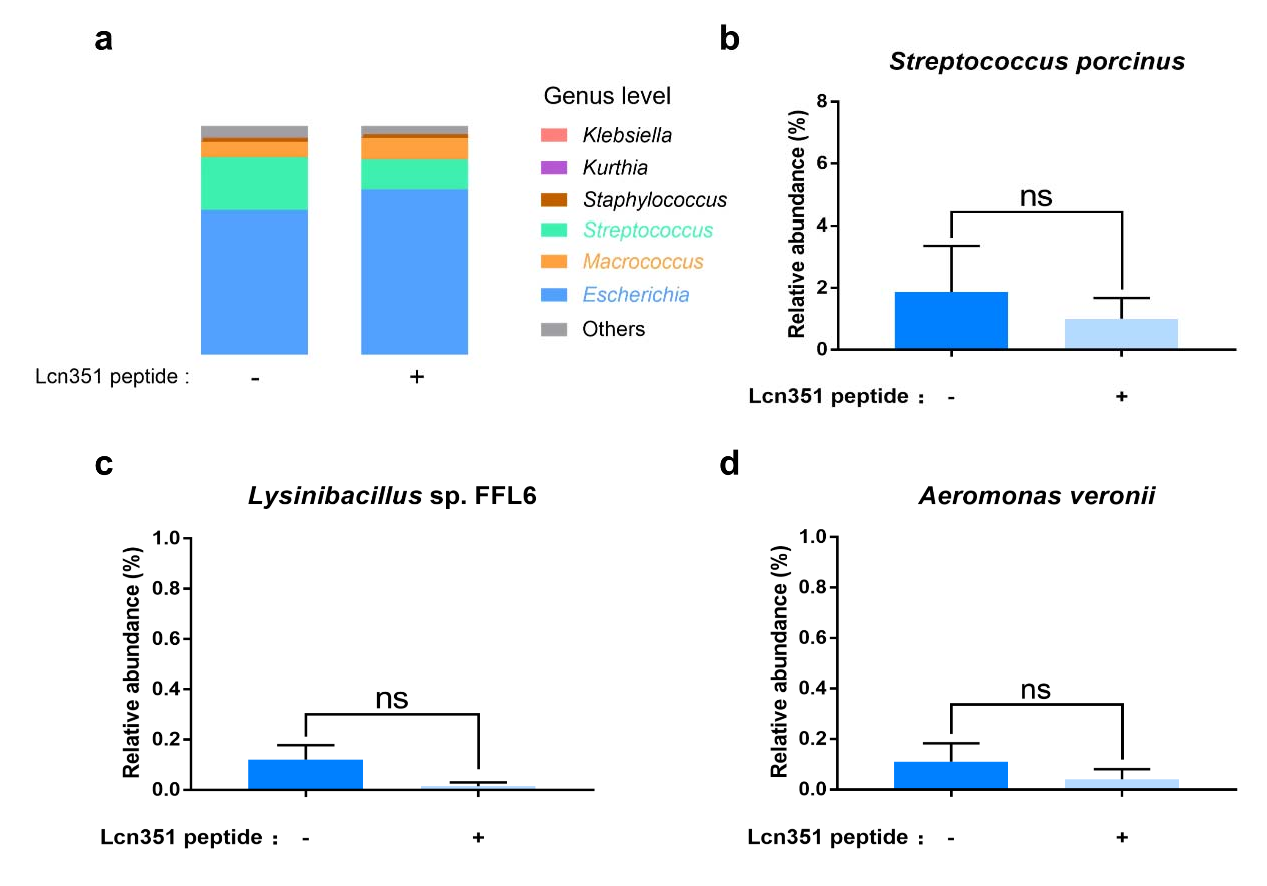


**Figure S6.** The effect of Lcn351 on the growth of other bacteria from pig tonsils. (a) The relative abundance of genera in the culture-dependent microbial community, with or without Lcn351. The genera whose relative abundance exceeded 1% in the control group (-) are presented. The relative abundances of *S. porcinus* (b), *Lysinibacillus sp.* FFL6 (c), and *Aeromonas veronii* (d) in the culture-dependent community of pig tonsils are shown. The data in graphs represent the means ± SEM (n = 4), and ns indicate no significant difference by a two-tailed unpaired *t* test.
